# Search for *Campylobacter* spp. Reveals High Prevalence and Pronounced Genetic Diversity of Arcobacter butzleri in Floodwater Samples Associated with Hurricane Florence in North Carolina, USA

**DOI:** 10.1128/AEM.01118-20

**Published:** 2020-10-01

**Authors:** Jeffrey A. Niedermeyer, William G. Miller, Emma Yee, Angela Harris, Ryan E. Emanuel, Theo Jass, Natalie Nelson, Sophia Kathariou

**Affiliations:** a North Carolina State University, Department of Food, Nutrition and Bioprocessing Sciences, Raleigh, North Carolina, USA; b Produce Safety and Microbiology Research Unit, Agricultural Research Service, U.S. Department of Agriculture, Albany, California, USA; c North Carolina State University, Department of Civil, Construction and Environmental Engineering, Raleigh, North Carolina, USA; d North Carolina State University, Department of Forestry and Environmental Resources, Raleigh, North Carolina, USA; e North Carolina State University, Department of Biological and Agricultural Engineering, Raleigh, North Carolina, USA; Centers for Disease Control and Prevention

**Keywords:** *Arcobacter*, *Arcobacter butzleri*, *Campylobacter*, *Campylobacter jejuni*, floodwaters, hurricane, MLST, genotype

## Abstract

In September 2018, Hurricane Florence caused extreme flooding in eastern North Carolina, USA, a region highly dense in concentrated animal production, especially swine and poultry. In this study, floodwater samples (*n* = 96) were collected as promptly post-hurricane as possible and for up to approximately 30 days and selectively enriched for *Campylobacter* using Bolton broth enrichment and isolation on modified charcoal cefoperazone deoxycholate agar (mCCDA) microaerobically at 42°C. Only one sample yielded *Campylobacter*, which was found to be Campylobacter jejuni with the novel sequence type 2866 (ST-2866). However, the methods employed to isolate *Campylobacter* readily yielded *Arcobacter* from 73.5% of the floodwater samples. The *Arcobacter* isolates failed to grow on Mueller-Hinton agar at 25, 30, 37, or 42°C microaerobically or aerobically but could be readily subcultured on mCCDA at 42°C microaerobically. Multilocus sequence typing of 112 isolates indicated that all were Arcobacter butzleri. The majority (85.7%) of the isolates exhibited novel sequence types (STs), with 66 novel STs identified. Several STs, including certain novel ones, were detected in diverse waterbody types (channel, isolated ephemeral pools, floodplain) and from multiple watersheds, suggesting the potential for regionally dominant strains. The genotypes were clearly partitioned into two major clades, one with high representation of human and ruminant isolates and another with an abundance of swine and poultry isolates. Surveillance of environmental waters and food animal production systems in this animal agriculture-dense region is needed to assess potential regional prevalence and temporal stability of the observed A. butzleri strains as well as their potential association with specific types of food animal production.

**IMPORTANCE** Climate change and associated extreme weather events can have massive impacts on the prevalence of microbial pathogens in floodwaters. However, limited data are available on foodborne zoonotic pathogens such as *Campylobacter* or *Arcobacter* in hurricane-associated floodwaters in rural regions with intensive animal production. With a high density of intensive animal production as well as pronounced vulnerability to hurricanes, eastern North Carolina presents unique opportunities in this regard. Our findings revealed widespread incidence of the emerging zoonotic pathogen Arcobacter butzleri in floodwaters from Hurricane Florence. We encountered high and largely unexplored diversity while also noting the potential for regionally abundant and persistent clones. We noted pronounced partitioning of the floodwater genotypes into two source-associated clades. The data will contribute to elucidating the poorly understood ecology of this emerging pathogen and highlight the importance of surveillance of floodwaters associated with hurricanes and other extreme weather events for *Arcobacter* and other zoonotic pathogens.

## Abstract (precis)

Climate change and associated extreme weather events can have massive impacts on the prevalence of microbial pathogens in floodwaters. However, limited data are available on foodborne zoonotic pathogens such as *Campylobacter* or *Arcobacter* in hurricane-associated floodwaters in rural regions with intensive animal production. With a high density of intensive animal production as well as pronounced vulnerability to hurricanes, eastern North Carolina presents unique opportunities in this regard. Our findings revealed widespread incidence of the emerging zoonotic pathogen Arcobacter butzleri in floodwaters from Hurricane Florence. We encountered high and largely unexplored diversity while also noting the potential for regionally abundant and persistent clones. We noted pronounced partitioning of the floodwater genotypes into two source-associated clades. The data will contribute to elucidating the poorly understood ecology of this emerging pathogen and highlight the importance of surveillance of floodwaters associated with hurricanes and other extreme weather events for *Arcobacter* and other zoonotic pathogens.

## INTRODUCTION

Hurricanes and other extreme weather events that can result in massive flooding of urban or agricultural areas have profound public health implications for contamination of surface waters (1–10). Chemical contaminants (e.g., heavy metals, antibiotics, and other pharmaceuticals) can leak from overflowing, inundated, or damaged sewage or animal waste containment structures into adjacent surface waters. Microbial agents, including pathogenic bacteria, viruses, and parasites, can similarly become introduced into surface waters and persist on agricultural land and in urban areas. Such hazards are accentuated in rural areas with concentrated animal production, including concentrated animal feeding operations. However, relevant data remain sparse and incomplete, primarily due to impaired accessibility, safety considerations, and accompanying delays in accessing and sampling impacted sites. There is a notable lack of reports that assess hurricane impacts on biological and chemical contaminants in floodwaters and in the context of geospatial features. Observational data gaps related to microbial water quality in floodwaters have prevented investigation of questions related to the importance of dilution relative to increased exposure. Although flooding increases potential exposure of surface waters to microbes, the large volumes of water associated with flooding may also dilute microbial agents, in turn counteracting the effects of increased contaminant loading.

On 12 September 2018, Florence, a large, slow-moving hurricane, made landfall on the North Carolina coast, resulting in record-breaking flooding for several locations. In the 7 days that followed, certain North Carolina communities received over 30 in. of rain, surpassing any of the previously recorded amounts of rainfall from a single storm in the region and resulting in unprecedented flood magnitudes for many inland rivers (11). Such heavy rainfall and flooding can massively impact water quality and safety in flooded areas, especially via runoff from agricultural operations. Eastern North Carolina is highly dense in facilities that produce food animals, including swine and poultry, especially turkeys (12, 13). Swine production units with multiple houses and large numbers of animals in each house are highly prevalent in the region, with turkey and swine production frequently interspersed (14). Animal production is a leading source of employment for many of the region’s residents. However, this region is also prone to a high frequency of severe weather events, including major hurricanes (15).

Hurricane Florence was preceded 2 years earlier by another major hurricane (Matthew, 28 September to 10 October 2016) with long-lasting adverse impacts on the socioeconomic landscape of North Carolina. Several of the Hurricane Florence-impacted areas had been previously flooded by Matthew. A research team in North Carolina had been assembled to investigate the environmental and public health impacts of Hurricane Matthew (16). Therefore, this team was already in place and readily poised to collect and analyze Hurricane Florence-associated floodwater samples as soon as it became logistically possible and safe to reach impacted areas. The original objective of the current study was to assess the prevalence of Campylobacter jejuni and Campylobacter coli in the floodwaters and allow comparisons with genotypic data collected over several years of investigation of these zoonotic pathogens in food animals and wildlife in this region (14, 17–25). However, in the course of the study, we detected numerous samples positive for *Arcobacter*, and therefore, we undertook the additional objective of characterizing the prevalence and genotypic diversity of *Arcobacter* from the hurricane-associated floodwaters.

## RESULTS

### *Campylobacter* was rarely detected in the floodwater samples, which instead frequently yielded *Arcobacter*.

Of the 98 water samples from the hurricane-impacted watersheds (96 from the floodwaters in phases 1 and 2, 2 from the Lumbee basin 3 weeks later), only 1 (1.0%), a sample of channel water from the Waccamaw watershed in phase 2, was positive for *Campylobacter*. Several putative *Campylobacter* colonies from this sample were purified on Mueller-Hinton agar (MHA), and all were found to be Campylobacter jejuni. Multilocus sequence typing (MLST) analysis of two of these isolates revealed a novel sequence type (ST), ST-2866 (see Table S1 in the supplemental material).

Interestingly, the enrichment procedures employed for *Campylobacter* yielded *Campylobacter*-like organisms from a large portion (72/98; 73.5%) of the samples. On modified charcoal cefoperazone deoxycholate agar (mCCDA), these *Campylobacter*-like cultures had colony appearance suggestive of *Campylobacter*, and helical, motile cells were noted with phase-contrast microscopy. However, unlike *Campylobacter* spp., these organisms grew poorly or not at all upon subculture on MHA or on tryptic soy agar with 5% sheep blood (Remel Microbiology Products, Lenexa, KS) and incubation at either 42 or 37°C microaerobically but could be readily subcultured on mCCDA at 42°C microaerobically. Sequencing of PCR products obtained from a subset of isolates using 16S rRNA gene primers indicated 99% identity with Arcobacter butzleri. The genus *Arcobacter* has been proposed to be reorganized into five novel genera, one of which, *Aliarcobacter* gen. nov., would include the species currently designated as Arcobacter butzleri (26). However, as discussed elsewhere (27), we consider the designation *Arcobacter* (for “aerotolerant campylobacters”) valid, pending a thorough phylogenomic assessment of *Epsilonproteobacteria* that would include *Campylobacter*, *Helicobacter*, and other genera, and therefore have chosen to maintain this taxonomic designation in this work.

**FIG 1:**
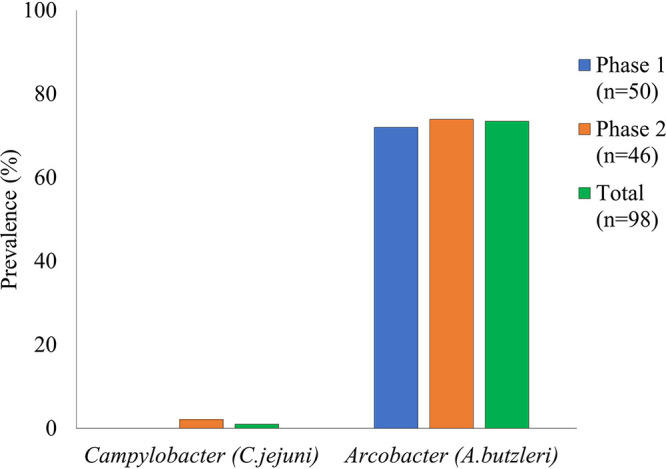
Prevalence of *Campylobacter* and *Arcobacter* in Hurricane Florence-impacted watershed samples over the study period. Sample collection and processing for *Campylobacter* and *Arcobacter* were performed as described in Materials and Methods.

**FIG 2:**
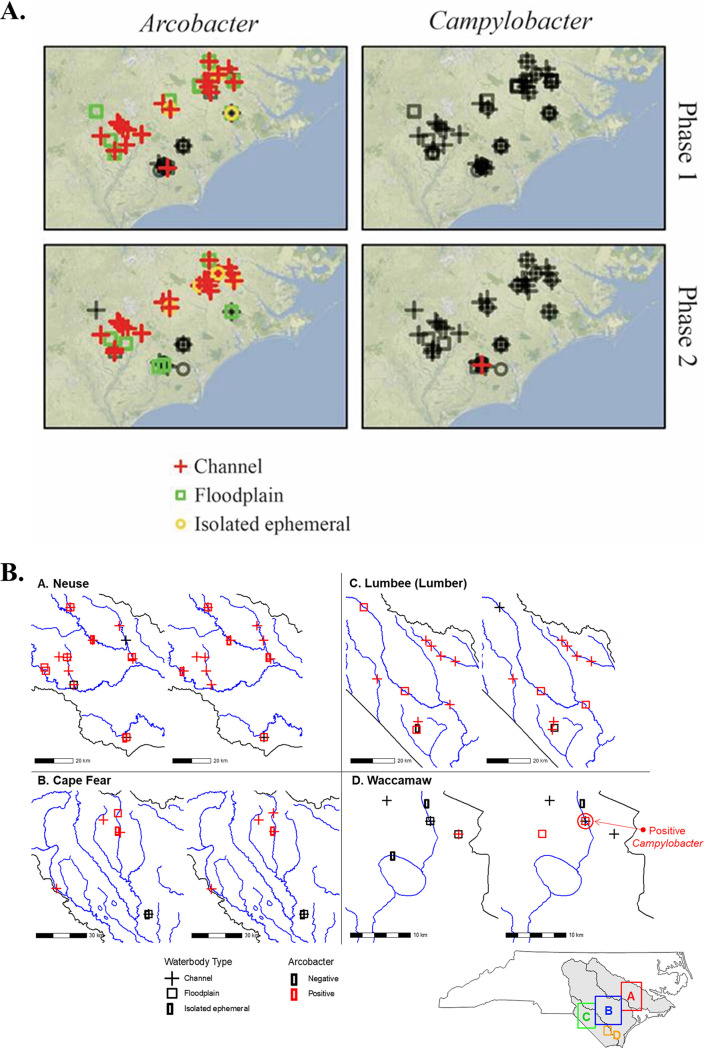
Water types and sampling sites. (A) Sample results for *Campylobacter* and *Arcobacter* over the two sampling phases are indicated in red (positive) and black (negative), and waterbody types are shown by the indicated symbols. Base map tile was from Stamen (terrain style), with open-source data from OpenStreetMap and OpenStreetMap Foundation. Maps were created in R using the ggmap package. (B) Distribution of the sampling sites by watershed as follows: Neuse River Basin (A), Cape Fear River Basin (B), Lumbee (Lumber) River Basin (C), and Waccamaw Basin, a sub-basin of the Lumbee Basin (D). Samples positive and negative for *Arcobacter* are shown in red and black, respectively, and waterbody types are shown by the indicated symbols. The sole *Campylobacter*-positive sample site is also indicated on the map. The blue lines correspond to major hydrographic features, and the gray shaded areas correspond to the river basins. Scale bars (in km) are included for maps A to D, and the location of the four watersheds in the reference map of the state of North Carolina is shown at the bottom right of the figure. The map was created with R using open-source geospatial hydrography data accessed through the North Carolina Department of Environmental Quality (http://data-ncdenr.opendata.arcgis.com/datasets/major-river-basins). The Lumbee river designation is in accordance with an ordinance passed by the Lumbee Tribal Council calling on all parties to observe the river’s ancestral name. County, state, and federal government utilize the designation “Lumber river,” created by state legislation in the 19th century (48, 49).

Putative *Arcobacter* was recovered frequently from enrichments of either water suspensions or filters (63.1 and 79.7%, respectively). Of the 65 samples for which both water suspensions and filters were enriched, 64.1% were positive for *Arcobacter* for both water and filters, while 3.1 and 16.9% were positive only with the water suspension or the filter, respectively. Prevalences of *Arcobacter* were similar in samples from phase 1 and phase 2 (72.0 and 73.9%, respectively) (Fig. 1). In each phase, the *Arcobacter*-positive samples were distributed throughout the sampling region without any noticeable spatial clustering within each sampled watershed and were recovered with similar frequency from samples of the two most prevalent waterbody types, i.e., channel (42/54; 77.8%) and floodplain (19/25; 76.0%) (Fig. 2). Total prevalence of *Arcobacter* across the two sampling phases was similar in the Neuse and Lumbee watersheds (34/39 [87.2%] and 21/24 [87.5%]) followed by the Cape Fear basin (12/20; 60.0%) and Waccamaw sub-basin (3/13; 23.1%).

### Arcobacter butzleri from floodwater samples exhibited high genotypic diversity, with several genotypes isolated from multiple floodwater types, watersheds, and sampling time points.

**FIG 3:**
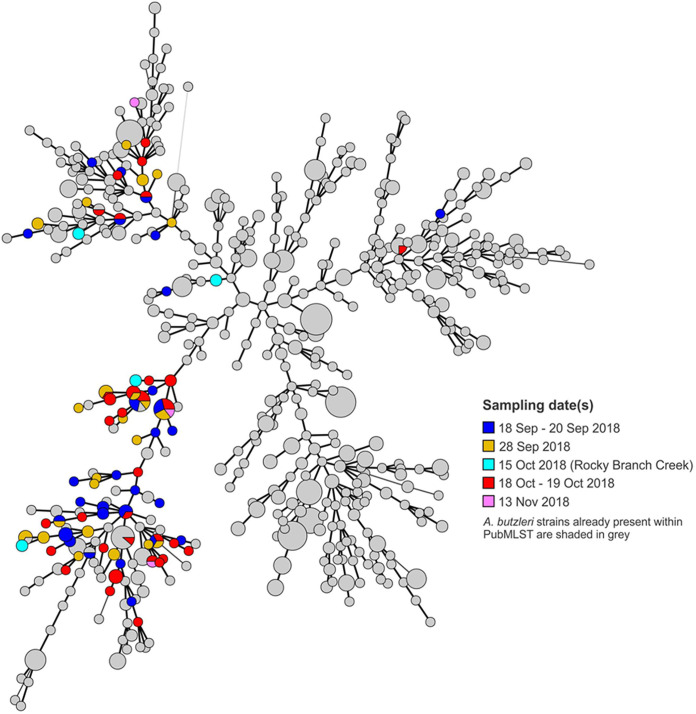
Genotype distribution of A. butzleri floodwater isolates in the different sampling periods. The MLST-based minimum spanning tree demonstrates the genotype distributions of A. butzleri isolated and genotyped in this study (in colors other than gray) and all other A. butzleri in the A. butzleri PubMLST database (gray). Each circle represents a different ST determined by MLST. The size of the circle indicates the number of isolates with the corresponding ST, with the smallest circles corresponding to one isolate. Closely related STs are connected by thick black lines. Phase 1 (blue and gold), 18 to 28 September 2018; phase 2 (red), 18 to 19 October 2018. Genotypes of isolates from Rocky Branch Creek on 15 October 2018 are in turquoise. Genotypes of isolates from two additional samples of the Lumbee watershed collected on 13 November 2018 are in pink. MLST analysis and minimum spanning tree construction were done as described in Materials and Methods.

**FIG 4:**
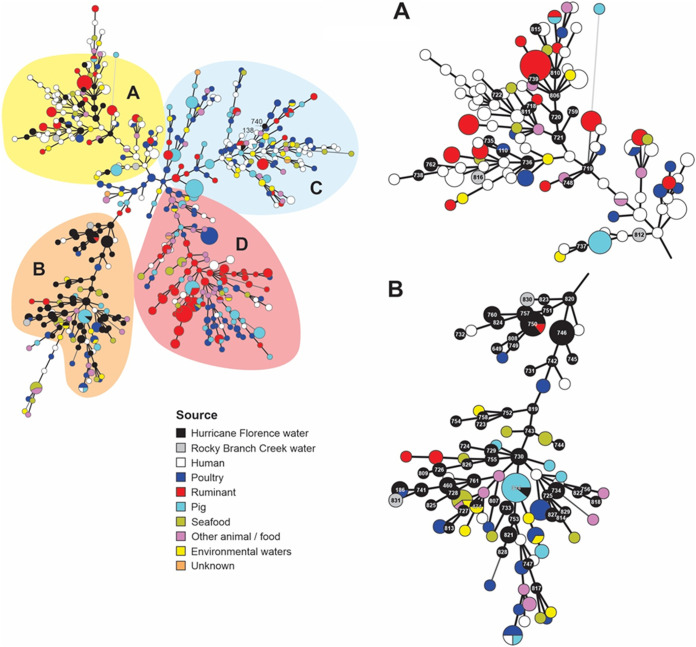
Relatedness of A. butzleri floodwater isolates to A. butzleri isolates from different sources. The MLST-based minimum spanning tree demonstrates the genotype distributions of A. butzleri isolated and genotyped in this study in the context of all other A. butzleri from diverse sources available in the A. butzleri PubMLST database. Florence floodwater and Rocky Branch Creek isolates are in black and gray, respectively, and other sources are in various other colors, as indicated in the inset. Major identified clades A, B, C, and D are indicated, with A and B harboring all but two of this study’s genotypes, exhibited in higher resolution on the right-hand side of the figure. Numerical ST designations of the floodwater isolates are indicated inside the circles in each cluster (A and B) inset on the right. The two STs (ST-138 and ST-740) outside of panels A or B are shown in cluster C (left-hand side). Each circle represents a different MLST-based ST. The size of the circle indicates the number of isolates with the corresponding ST, with the smallest circles corresponding to one isolate. Closely related STs are connected by thick black lines. MLST analysis and minimum spanning tree construction was performed as described in Materials and Methods.

MLST analysis of 112 putative *Arcobacter* isolates confirmed that all were A. butzleri and identified 74 STs, of which 66 were novel (see Table S1). The novel STs accounted for the majority (96/112; 85.7%) of the isolates that were genotyped. Most of these novel STs were encountered just once among the floodwater isolates, but several were detected in isolates from multiple samples (Fig. 3; see also Table S1). Even though different colonies from the same enrichment typically had the same ST, different STs were frequently identified in suspension versus filter enrichments of the same sample (Table S1). Among the previously identified STs, we identified some that were shared with isolates of swine (ST-314), environmental water (ST-474), ruminant (ST-138 and ST-750), poultry (ST-110 and ST-186), and human origin (ST-138) (Fig. 4).

Several (*n* = 14) STs, of which all but two were novel, were identified in isolates from different samples (Table 1). On several occasions, the same ST was identified in different water types, time points, and watersheds. Only two of these STs (ST-757 and ST-760, both novel) were encountered in just one watershed (Neuse), each at different times during the sampling period (Table 1). Of the remainder, most were from the Lumbee and at least one additional watershed, with two (ST-821 and ST-834) recovered exclusively from watersheds other than the Lumbee. Certain STs detected in 3 or more samples were noteworthy in their distribution. For instance, the novel ST-730 was identified in ephemeral water bodies and channel water on two different dates spanning 1 month and in both the Cape Fear and Lumbee watersheds. ST-746, also novel, was isolated from channel samples in all four watersheds across the two sampling phases, spanning an entire month. ST-734 and ST-750 were found in ephemeral, floodplain, and channel samples from the Lumbee as well as the Neuse watersheds on three different dates, again spanning a whole month (Table 1 and Fig. 3).

**TABLE 1:** A. butzleri STs identified in multiple samples

ST (no. of samples, ST cluster)^*a*^	Date (no. of samples)^*b*^	Waterbody type (no. of samples)^*c*^	Watershed (no. of samples)^*d*^
**721** (2, A)	18 September (1); 18 October (1)	1 (2)	L (1); N (1)
**736** (2, A)	18 September (1); 18 October (1)	1 (1); 2 (1)	L (1); N (1)
460 (3, B)	18 September (3)	1 (2); 4 (1)	L (2); CF (1)
**726** (2, B)	18 September (2)	1 (1); 2 (1)	L (1); CF (1)
**729** (2, B)	18 September (2)	1 (1); 3 (1)	L (1); CF (1)
**730** (3, B)	18 September (2); 18 October (1)	1 (1); 3 (2)	L (1); CF (2)
**734** (3, B)	18 September (2); 18 October (1)	1 (1); 2 (1); 3 (1)	L (1); N (2)
**746** (6, B)	18 September (2); 28 September (1); 18 October (2); 13 November (1)	1 (5); LB (1)	L (3); CF (1); N (1); W (1)
750 (5, B)	18 September (2); 28 September (1); 18 October (2)	1 (1); 2 (3); 3 (1)	L (1); N (4)
**757** (2, B)	18 September (1); 18 October (1)	1 (1); 3 (1)	N (2)
**760** (2, B)	28 September (1); 18 October (1)	1 (2)	N (2)
**821** (2, B)	18 October (1); 19 October (1)	2(1); 3 (1)	CF (1); W (1)
**824** (2, B)	18 October (2)	2 (1); NA (1)	CF (1); N (1)
**827** (2, B)	18 October (1); 13 November (1)	2 (1); LB (1)	L (2)

a Novel STs are in bold font. Clusters are as in Table S1 in the supplemental material and Fig. 4.

b Dates are all in the year 2018.

c Waterbody types, as in Table S1. 1, channel; 2, floodplain; 3, isolated ephemeral; 4, other (large pond); NA, information not available; LB, Lumbee Basin, collected post-phase 2 on 11 November 2018.

d L, Lumbee; N, Neuse; CF, Cape Fear; W, Waccamaw. Detailed information on the coordinates of the samples is present in Table S1.

### Floodwater A. butzleri genotypes partitioned in two major clades with different source-associated compositions.

All but two of the 112 A. butzleri STs from the floodwater isolates were partitioned in two major clades, designated clusters A and B (Fig. 4). The exceptions were ST-138 and ST-740, which were localized in a different clade, designated cluster C (Fig. 4). The majority of floodwater isolates grouped in cluster B (88/112; 78.6%) followed by cluster A (22/112; 19.6%). Source distribution analysis including the other STs available in the A. butzleri PubMLST database revealed that cluster A was highly populated by isolates of human and ruminant origin, with notable underrepresentation of poultry- or swine-derived isolates (Fig. 4; see also Table S2 in the supplemental material). The opposite was found in cluster B, where floodwater isolates were closely related to others of poultry and swine origin (Fig. 4; see also Table S2). Only 1 of the 112 genotyped floodwater isolates, from cluster B, shared its ST (ST-474) with an isolate previously obtained from environmental water (Fig. 4; see also Table S2). Isolates from environmental water (outside of the floodwater isolates in the current study) were relatively uncommon in either cluster and were mostly found in cluster C (Fig. 4; Table S2), which included two of the floodwater STs (ST-138 and ST-740) from the current study. Isolates of human and food animal origin were also well represented in cluster C (Fig. 4; Table S2). None of the floodwater isolates from the current study mapped within another major cluster (designated D in Fig. 4), which included multiple STs from foods and food animals (Fig. 4).

Even though certain repeatedly encountered STs were isolated from multiple watersheds and waterbody types (Table 1), cluster composition suggested potential dependence on watershed. Isolates from the Cape Fear watershed composed similar portions of both clusters A and B (13.6 and 15.9%, respectively), similar to those from the Lumbee watershed (27.3 and 30.7%, respectively). However, more disproportionate contributions to clusters A and B were noted for isolates from the Neuse watershed (59.1% and 46.6%, respectively). Furthermore, Waccamaw isolates, albeit relatively few (*n* = 6 with three different novel STs), were all found in cluster B, making up approximately 6.8% of the floodwater isolates in that cluster. Both STs in cluster C were from the Neuse watershed.

**FIG 5:**
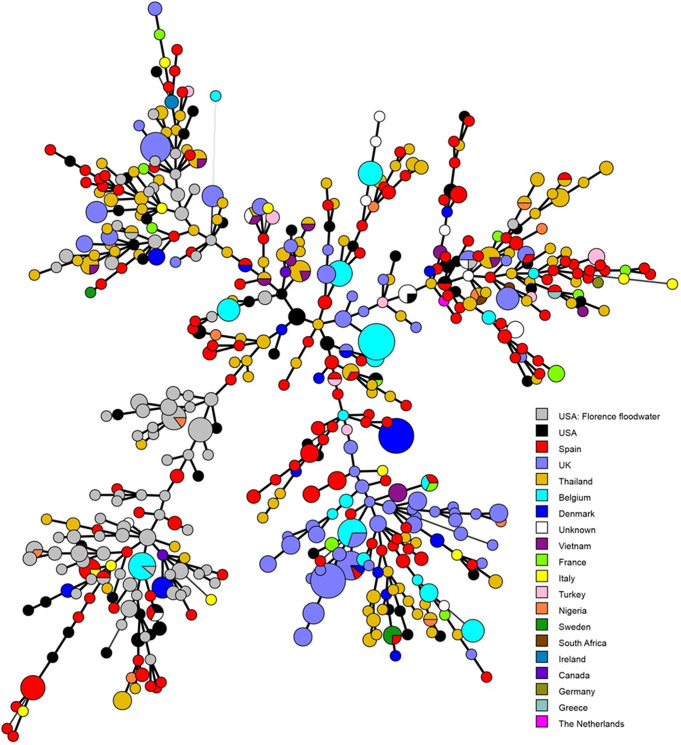
Relatedness of A. butzleri floodwater isolates to A. butzleri isolates from different countries. The MLST-based minimum spanning tree demonstrates the genotype distributions of the floodwater isolates in the context of A. butzleri from different countries. A. butzleri isolated and genotyped in this study are in gray, while other isolates from the United States are in black. Diverse colors are used for other countries, as shown in inset. Included are all A. butzleri isolates in the A. butzleri PubMLST database. Each circle represents a different MLST-based ST. The size of the circle indicates the number of isolates with the corresponding ST, with the smallest circles corresponding to one isolate. Closely related STs are connected by thick black lines. U.S. isolates previously outside of those in the current study are in black.

Geographically, cluster A consisted mostly of isolates from the United States (many from the current study), Thailand (primarily human), and the United Kingdom (primarily ruminant) (Fig. 4 and 5; see also Table S2). In contrast, cluster B had a significant representation of isolates from the United States (primarily from the current study) and from Spain (primarily poultry, seafood, and other foods) (Fig. 4 and 5; see also Table S2). U.S. isolates outside of the current study tended to be of human origin (Fig. 4 and 5; see also Table S2).

## DISCUSSION

Even though surface water is considered a major source of pathogens that can contaminate the food supply, little is known about the prevalence or genotypes of *Campylobacter* and *Arcobacter* in floodwaters associated with extreme weather events such as hurricanes. Moreover, the lack of data on pathogen presence in floodwaters limits our understanding of whether floodwaters have high microbial contaminant concentrations due to increased surface water contact with contaminant sources or if the large volumes of water associated with floodwaters ultimately dilute microbial agents and result in low contaminant concentrations. Consequently, estimates of public health risks associated with surface waters in flood and post-flood conditions remain imprecise. The current study is, to our knowledge, the first report on the prevalence and genotypes of *Campylobacter* and *Arcobacter* in hurricane-associated floodwaters. Our findings suggested that *Campylobacter* was uncommon (only one sample, 1.0%), while the methods employed for *Campylobacter* yielded *Arcobacter* from the majority (73.5%) of the samples.

As indicated above, reports on *Arcobacter* prevalence in hurricane-associated floodwaters have been lacking. However, *Arcobacter* contamination of groundwater subsequent to extreme precipitation events was previously implicated in a massive waterborne outbreak in the Lake Erie region (28). Of the 16 groundwater wells surveyed in that study, 7 were found to be positive for *Arcobacter. Campylobacter* was not detected, but *Arcobacter* spp. were recovered on the selective media employed for *Campylobacter* (28), as was also the case in our study. Unfortunately, the species or genotypes of *Arcobacter* involved in that outbreak and groundwater contamination were not determined (28).

In our study, all analyzed *Arcobacter* isolates were found to be A. butzleri, an emerging waterborne pathogen which has also been repeatedly isolated from diverse types of food (29–37). The media and relatively high temperature employed here (42°C) may well have prevented the recovery of other *Arcobacter* species that may have been present in the floodwaters. In several studies, however, A. butzleri was one of the most commonly isolated *Arcobacter* species from water sources (35, 38–41). The prevalence of *Arcobacter*-positive samples in our study was high (73.5%), even though the selective media and conditions were those intended for *Campylobacter*. A previous study utilizing *Arcobacter*-specific selective media reported a similar prevalence (75%) from a river catchment in Spain in autumn and winter, with higher prevalence in the spring and summer (41). Analysis of river, sewage water, and spring water in Turkey, utilizing *Arcobacter*-specific media, revealed *Arcobacter* prevalences of 52, 36.4, and 12.5%, respectively (40). Our study might have revealed an even higher prevalence had *Arcobacter*-specific media been employed. In a previous study, however, rigorous analysis of a panel of A. butzleri strains for growth on mCCDA versus cefoperazone amphotericin teicoplanin (CAT) agar designed for *Arcobacter* indicated that all strains could grow on mCCDA, even though some grew better on CAT (42). In future studies, use of both mCCDA and CAT or other *Arcobacter*-specific media will be valuable to maximize the chances of recovery of both *Campylobacter* and *Arcobacter* spp. from floodwaters.

The identification of 74 STs among the 112 A. butzleri isolates that were genotyped from the floodwaters suggests a highly diverse population. The majority (66/74; 89.2%) of the STs from the hurricane-impacted watershed samples were novel. This may reflect the fact that the A. butzleri PubMLST database is still underpopulated. In comparison to *Campylobacter* species such as C. jejuni and C. coli, A. butzleri and other *Arcobacter* spp. remain much less commonly investigated and genotyped. However, the findings may also reflect regional diversity in the population that we analyzed. The A. butzleri PubMLST database lacked isolates from the same region as the floodwater isolates investigated here, i.e., eastern and southeastern North Carolina.

The fact that several dominant STs (e.g., ST-460, ST-730, ST-734, ST-746, and ST-750) (Table 1) were encountered in isolates from multiple sample types, watersheds, and time points that on certain occasions spanned the entire month of sampling may reflect regionally prevalent strains of A. butzleri. The repeated detection of the novel ST-757 and ST-760 in only one watershed (Neuse) may reflect localized prevalence of the corresponding strains in that watershed, a speculation that will need to be addressed by further sampling in the Neuse and other watersheds. Conversely, the repeated detection of several STs in multiple watersheds that lacked surface connectivity (Table 1) suggests widespread distribution of the corresponding strains in the region. In this context, it is of interest that A. butzleri strains isolated during the same time period from Rocky Branch Creek, an urban creek in Raleigh, NC, had STs that differed from those recovered from the floodwater samples but were still localized in clusters A and B (2 STs each) (Fig. 4). Continued analysis of A. butzleri from environmental waters will be critical to elucidate the geographic and temporal distribution of the strains encountered in the floodwater samples in order to better understand transmission dynamics and inform management and mitigation strategies.

Previously identified STs in the floodwater isolates from this study were shared with isolates of swine, poultry, or ruminants from other countries. We currently lack information on the prevalence or genotypes of A. butzleri in agricultural animals in the Hurricane Florence-impacted region. Such information is needed to determine whether apparently dominant and persistent A. butzleri STs identified in the floodwaters, e.g., ST-746 and ST-750, may also be prevalent in animals produced in this food animal-dense region or in surface waters during nonflooded conditions.

Previous studies of turkey and swine farms in eastern North Carolina as well as wildlife and cattle in the same region, using the same culture conditions as those employed here, revealed a high prevalence of *Campylobacter*; *Arcobacter* was not isolated from those samples, which yielded exclusively C. jejuni or C. coli (14, 17–19, 21, 24, 25). In the floodwater samples analyzed in the current study, *Campylobacter* prevalence was low (1.0%) in contrast to the overall high prevalence (73.5%) of *Arcobacter*. Even though this may be due to true scarcity of *Campylobacter*, especially considering the relatively low volume of water that was analyzed, it may also reflect preferential recovery of *Arcobacter* from water samples that may be contaminated with both *Arcobacter* and *Campylobacter*, or possibly higher relative fitness of *Arcobacter* in these samples. Major gaps currently exist in our understanding of the relative fitness of *Arcobacter* and *Campylobacter* in environmental water and feces from agricultural animals.

In conclusion, our analysis of Hurricane Florence-associated floodwater samples revealed that *Campylobacter* was uncommon, with C. jejuni detected only once, while *Arcobacter*, specifically the emerging waterborne pathogen A. butzleri, was frequently recovered employing media and conditions intended for *Campylobacter*. Genotyping via MLST revealed high genotypic diversity among the A. butzleri isolates and a multitude of novel STs. Several STs, including novel ones, were detected in multiple watersheds, diverse types of water (channel, isolated ephemeral pools, floodplain), and repeatedly over the project survey period, suggesting the potential for dominant, persistent clones. Genotyping clearly partitioned the floodwater-associated A. butzleri isolates into two major clades, one of which had high representation of human and ruminant isolates, while the other was highly populated by swine and poultry isolates. The phylogenetic relationships among these strains and their relatedness among themselves and those from other sources will be enhanced by continued surveillance and higher-resolution genotyping as may be allowed with whole-genome sequencing, which is currently being undertaken for the floodwater-derived A. butzleri strains. Such information will need to be complemented by currently lacking data on prevalence or genotypes of A. butzleri in agricultural animals in the impacted region. The widespread prevalence of A. butzleri in floodwaters, despite the opportunity for dilution, may signal that surface waters pose risks to public health during flooded conditions. Further, given that samples were collected shortly after hurricane landfall and throughout the course of several weeks thereafter, results suggest that public health risks associated with surface waters may persist beyond the peak of flooding. Further work is needed to determine the prevalence and genotypes of *Arcobacter* and *Campylobacter* in the watersheds of this region during hurricane-associated flooding but also in the absence of severe weather events so that an assessment of baseline levels of *Arcobacter* can be made. Data are also needed on baseline incidence of human *Arcobacter* infections in this region and potential increases during hurricane-associated flooding. Such data are lacking. *Arcobacter* infections are currently not reportable and likely are rarely diagnosed, especially in the affected region, which is largely rural, low-income, and generally underserved, with a relative scarcity of clinics that would collect and analyze human diarrheal samples. All water samples were collected from water bodies in tier 1 counties, a designation reflecting highest distress levels based on economic well-being metrics (43). There is a critical need for integration of surveillance of environmental waters for pathogens such as *Arcobacter* and *Campylobacter* with public health data on the incidence of waterborne gastrointestinal illness in the hurricane-impacted communities.

## MATERIALS AND METHODS

### Water sample collection.

A total of 96 floodwater samples were collected at sites in the Neuse (*n* = 39), Cape Fear (*n* = 20), Lumbee (Lumber) (*n* = 24), and Waccamaw (a sub-basin of the Lumbee) (*n* = 13) watersheds in eastern North Carolina (Fig. 2; see also Table S1 in the supplemental material). The Neuse (Fig. 2B, panel A), Cape Fear (Fig. 2B, panel B), and Lumbee (Fig. 2B, panel C) are all distinct river basins, without surface connectivity between them. Water was collected in autoclaved one-liter Nalgene bottles triple-rinsed with the target sample water prior to collection. Flood sample collection sites were classified into the following four categories: (i) channel, i.e., flowing water in stream channels; (ii) floodplain, i.e., slow-moving or stagnant floodplain water; (iii) isolated ephemeral, e.g., pools of floodwater likely to dry within a few days in the absence of additional rainfall; and (iv) other, such as isolated permanent water bodies (i.e., ponds, lakes). Sampling was performed in two distinct time periods, designated phase 1 and phase 2, yielding 50 and 46 samples, respectively. Phase 1 sampling started within 7 days of Hurricane Florence’s landfall and occurred between 18 September 2018 and 28 September 2018, while phase 2 sampling occurred on 18 October 2018 and 19 October 2018. Coordinates of each sample location were recorded and logged using a handheld global positioning system (GPS) unit and Google Earth. Efforts were made to sample the same sites in both phases. In cases where the exact sample site from phase 1 was no longer available (i.e., area was no longer flooded), samples were collected from a nearby similar location. Additional samples were taken from the Lumbee watershed on 13 November 2018 (*n* = 2) and the Rocky Branch Creek, in Raleigh, NC, on 15 October 2018 (*n* = 4). Upon collection, the samples were immediately stored in coolers on ice, transported to the laboratory, and stored at 4°C until processing, typically within 24 to 72 h.

### Isolation of *Campylobacter* and *Arcobacter*.

The majority of the samples (*n* = 64) were processed via parallel enrichments of water (1.3 ml) as well as 0.45-μm filters (Thermo Fisher Scientific, Inc., Waltham, MA) prepared via vacuum filtration of 50 ml water, while 34 samples were processed only via enrichments of the water suspension. The filters were subsequently cut with flame-sterilized scissors into three equal-size fragments, one of which was used for enrichment of *Campylobacter* (the remaining fragments were utilized to enrich for Salmonella enterica and *Listeria* spp., which will be described in a separate presentation). The water samples and filter fragments were enriched for *Campylobacter* in 10 ml Bolton broth (Oxoid Ltd., Hampshire, UK) and incubated under microaerobic conditions at 37°C for 24 h using GasPak EZ Campy sachets (Becton, Dickinson and Co., Sparks, MD, USA). Following enrichment, appropriate dilutions were prepared, 100 μl was spread plated on modified charcoal cefoperazone deoxycholate agar (mCCDA; Oxoid), and the mCCDA plates were incubated microaerobically at 42°C for 48 h as described previously (17). An average of five putative *Campylobacter* colonies per positive sample were transferred from mCCDA to Mueller-Hinton agar (MHA; Becton, Dickinson and Co.) for purification following incubation microaerobically at 42°C for 48 h as described previously (17). Many cultures (found upon further analysis to be *Arcobacter*) grew poorly or not at all on MHA, and an average of four *Campylobacter*-like colonies from such cultures were purified via subculture on fresh mCCDA plates. Purified isolates were preserved at −80°C, and *Campylobacter* species designations were determined via multiplex PCR with *hip* and *ceu* primers to detect C. jejuni and C. coli, respectively, as described (17). A subset of isolates that did not yield an *hip* or *ceu* amplicon via multiplex PCR were analyzed via 16S rRNA sequencing (Genewiz, South Plainfield, NJ, USA) of the amplicon obtained from the universal 16S primers 8f (5′-AGA GTT TGA TCC TGG CTC AG-3′) (44) and 1492R (5′-GGT TAC CTT GTT ACG ACT T-3′) (45).

### Multilocus sequence typing and minimum spanning trees.

*Campylobacter* or *Arcobacter* isolates were chosen so as to represent each positive sample and enrichment type (suspension or filter) and characterized via multilocus sequence typing (MLST) as described (46, 47). Novel C. jejuni and A. butzleri alleles and sequence types were deposited into the corresponding PubMLST databases (https://pubmlst.org/campylobacter/; https://pubmlst.org/arcobacter/). Concatenated sequences representing all A. butzleri profiles within the PubMLST database, including those identified in this study, were downloaded from PubMLST on 14 November 2019 and again on 13 July 2020. These sequences were imported into BioNumerics (version 7.6.3; Applied Maths, Austin, TX) and aligned using the Fast algorithm. Within BioNumerics, a neighbor-joining dendrogram was constructed from the aligned profile sequences; minimum spanning trees (MSTs) were constructed based on the sequence distances between the concatenated profile sequences and using the default priority rules and “permutation resampling” strategy and “highscore summary” methods. MST nodes were color-coded within BioNumerics according to sampling date, source, or location of isolation.

### Sequence data.

All MLST sequence data have been deposited into PubMLST (https://pubmlst.org/campylobacter/; https://pubmlst.org/arcobacter/) as described above.

## Supplementary Material

Supplemental file 1
