# Supplementary material for: Search for Campylobacter spp. Reveals High Prevalence and Pronounced Genetic Diversity of Arcobacter butzleri in Floodwater Samples Associated with Hurricane Florence in North Carolina, USA
Source: Appl Environ Microbiol. 2020 Oct 1;86(20):e01118-20. doi: 10.1128/AEM.01118-20 (PMC7531973; doi:10.1128/AEM.01118-20)
Supplement: Supplemental file 1 [file AEM.01118-20-s0001.pdf]

Table S1: *Campylobacter jejuni* and *Arcobacter butzleri* from water samples analyzed in this study

'Cluster A' *Arcobacter butzleri* isolates

**Isolate[a]**	**2018 Sample Date (mo/d)**	**Phase**	**Source[b]**	**Latitude[c]**	**Longitude[c]**	**Water-shed[d]**	**Water Body Type[e]**	**Enrichment Type**	***aspA***	***atpA***	***glnA***	***gltA***	***glyA***	***pgm***	***tkt***	**ST[f]**
F79C-1A	10/18	2	HFW	35.380448	-77.86977	Neuse	1	Suspension	25	3	1	7	35	29	27	110
F9C-1A	9/18	1	HFW	35.1147	-78.4767	Cape Fear	1	Suspension	3	41	11	15	742	410	4	**718**
F49C-A	9/28	1	HFW	35.458232	-77.675775	Neuse	1	Suspension	3	41	11	20	54	70	31	**719**
F48C-A	9/28	1	HFW	35.369752	-77.446208	Neuse	1	Suspension	3	42	11	12	759	338	4	**720**
F48C-B	9/28	1	HFW	35.369752	-77.446208	Neuse	1	Suspension	3	42	11	12	759	338	4	**720**
F11C-1A	9/18	1	HFW	34.80137	-78.98528	Lumbee	1	Suspension	3	42	181	15	143	407	320	**721**
F69C-1A	10/18	2	HFW	35.313546	-77.944991	Neuse	1	Suspension	3	42	181	15	143	407	320	**721**
F22C-1A	9/18	1	HFW	35.046774	-78.366462	Cape Fear	1	Suspension	3	251	2	242	748	90	264	**722**
F27C-B	9/28	1	HFW	35.002593	-77.483371	Neuse	2	Suspension	25	30	1	246	35	29	27	**735**
F20C-2B	9/18	1	HFW	35.377817	-77.822484	Neuse	2	Filter	38	14	1	2	599	17	323	**736**
F65C-1A	10/18	2	HFW	34.52114	-79.17829	Lumbee	1	Suspension	38	14	1	2	599	17	323	**736**
F9C-2A	9/18	1	HFW	35.1147	-78.4767	Cape Fear	1	Filter	39	33	2	129	595	109	4	**737**
F6C-2A	9/18	1	HFW	35.378341	-77.451253	Neuse	2	Filter	40	66	113	20	751	101	4	**738**
F39C-B	9/28	1	HFW	34.55411	-79.17192	Lumbee	1	Suspension	57	14	26	12	756	408	19	**739**
F17C-2B	9/18	1	HFW	34.86359	-79.10484	Lumbee	1	Filter	292	30	127	239	754	48	20	**748**
F45C-B	9/28	1	HFW	35.525129	-77.522507	Neuse	1	Suspension	374	257	225	44	757	415	326	**759**
F53C-A	9/28	1	HFW	35.24874	-77.78456	Neuse	1	Suspension	377	41	226	22	765	238	328	**762**
F53C-B	9/28	1	HFW	35.24874	-77.78456	Neuse	1	Suspension	377	41	226	22	765	238	328	**762**
F59C-1A	10/18	2	HFW	34.86359	-79.10484	Lumbee	1	Suspension	3	30	1	15	769	421	27	**806**
F78C-1A	10/18	2	HFW	35.458324	-77.675567	Neuse	1	Suspension	31	42	11	15	768	338	330	**810**
F84C-1B	10/18	2	HFW	35.377912	-77.822425	Neuse	1	Suspension	39	3	2	34	74	35	200	**811**
RBC101518-3-A	10/15	N/A	RBC	N/A	N/A	N/A	RBC	Filter	39	5	1	34	775	109	38	**812**
RBC101518-3-B	10/15	N/A	RBC	N/A	N/A	N/A	RBC	Filter	39	5	1	34	775	109	38	**812**
F104C-1A	11/13	N/A	HFW	N/A	N/A	Lumbee	LB	Suspension	181	3	2	30	404	227	332	**815**
RBC101518-2-A	10/15	N/A	RBC	N/A	N/A	N/A	RBC	Filter	183	262	2	2	774	56	20	**816**
RBC101518-2-B	10/15	N/A	RBC	N/A	N/A	N/A	RBC	Filter	183	262	2	2	774	56	20	**816**

‘Cluster B’ *Arcobacter butzleri* isolates

Isolate[a]	2018 Sample Date (mo/d)	Phase	Source[b]	Latitude[c]	Longitude[c]	Water-shed[d]	Water-body Type[e]	Enrichment Type	*aspA*	*atpA*	*glnA*	*gltA*	*glyA*	*pgm*	*tkt*	ST[f]
F44C-A	9/28	1	HFW	35.612096	-77.804737	Neuse	1	Suspension	65	53	26	57	137	97	67	186
F44C-B	9/28	1	HFW	35.612096	-77.804737	Neuse	1	Suspension	65	53	26	57	137	97	67	186
F69C-2A	10/18	2	HFW	35.313546	-77.944991	Neuse	1	Filter	14	15	26	131	375	223	34	314
F21C-1B	9/18	1	HFW	34.88732	-79.13168	Lumbee	1	Suspension	234	15	26	164	375	260	176	460
F23C-1A	9/18	1	HFW	35.05744	-78.37756	Cape Fear	4	Suspension	234	15	26	164	375	260	176	460
F7C-2A	9/18	1	HFW	34.624032	-79.012825	Lumbee	1	Filter	234	15	26	164	375	260	176	460
F8C-2B	9/18	1	HFW	34.679601	-79.236498	Lumbee	2	Filter	81	62	128	148	119	244	183	474
F76C-2A	10/18	2	HFW	35.456645	-77.485555	Neuse	1	Filter	14	37	205	144	109	57	283	649
F49C-B	9/28	1	HFW	35.458232	-77.675775	Neuse	1	Suspension	5	33	26	62	761	416	327	**723**
F12C-2B	9/18	1	HFW	35.022618	-79.44241	Lumbee	2	Filter	5	62	224	55	753	409	204	**724**
F45C-A	9/28	1	HFW	35.525129	-77.522507	Neuse	1	Suspension	5	158	128	248	109	418	79	**725**
F13C-1A	9/18	1	HFW	35.1506	-78.3819	Cape Fear	2	Suspension	14	37	26	241	745	405	222	**726**
F39C-A	9/28	1	HFW	34.55411	-79.17192	Lumbee	1	Suspension	14	37	26	241	745	405	222	**726**
F51C-B	9/28	1	HFW	35.380448	-77.86977	Neuse	1	Suspension	14	49	26	55	763	414	32	**727**
F1C-1A	9/20	1	HFW	34.679601	-79.236498	Lumbee	2	Suspension	14	62	26	240	738	406	317	**728**
F1C-1B	9/18	1	HFW	34.679601	-79.236498	Lumbee	2	Suspension	14	62	26	240	738	406	317	**728**
F18C-1A	9/18	1	HFW	35.05744	-78.37756	Cape Fear	3	Suspension	14	151	222	144	739	409	76	**729**
F3C-1A	9/18	1	HFW	34.729631	-79.375567	Lumbee	1	Suspension	14	151	222	144	739	409	76	**729**
F18C-2A	9/18	1	HFW	35.05744	-78.37756	Cape Fear	3	Filter	14	253	26	144	755	223	322	**730**
F21C-2A	9/18	1	HFW	34.88732	-79.13168	Lumbee	1	Filter	14	253	26	144	755	223	322	**730**
F87C-1A	10/18	2	HFW	35.05744	-78.37756	Cape Fear	3	Suspension	14	253	26	144	755	223	322	**730**
F27C-A	9/28	1	HFW	35.002593	-77.483371	Neuse	2	Suspension	14	254	26	245	42	404	79	**731**
F28C-A	9/28	1	HFW	35.002593	-77.483371	Neuse	3	Suspension	14	255	50	40	174	122	79	**732**
F43C-A	9/28	1	HFW	35.612725	-77.804619	Neuse	2	Suspension	14	256	26	131	375	223	34	**733**
F43C-B	9/28	1	HFW	35.612725	-77.804619	Neuse	2	Suspension	14	256	26	131	375	223	34	**733**
F17C-1A	9/18	1	HFW	34.86359	-79.10484	Lumbee	1	Suspension	17	62	223	40	746	43	321	**734**
F55C-A	9/28	1	HFW	35.248663	-77.784804	Neuse	2	Suspension	17	62	223	40	746	43	321	**734**
F71C-2A	10/18	2	HFW	35.458092	-77.675781	Neuse	3	Filter	17	62	223	40	746	43	321	**734**
F47C-A	9/28	1	HFW	35.379864	-77.450886	Neuse	2	Suspension	65	53	26	249	638	97	67	**741**
F47C-B	9/28	1	HFW	35.379864	-77.450886	Neuse	2	Suspension	65	53	26	249	638	97	67	**741**
F2C-2A	9/18	1	HFW	34.823055	-79.057089	Lumbee	1	Filter	209	61	26	171	375	254	321	**742**
F7C-1A	9/18	1	HFW	34.624032	-79.012825	Lumbee	1	Suspension	212	149	26	144	453	38	183	**743**
F14C-1A	9/18	1	HFW	35.330005	-77.952903	Neuse	2	Suspension	212	149	26	144	469	38	222	**744**
F14C-2A	9/18	1	HFW	35.330005	-77.952903	Neuse	2	Filter	241	33	26	53	117	254	317	**745**
F105C-2A	11/13	N/A	HFW	N/A	N/A	Lumbee	LB	Filter	241	222	26	244	745	254	317	**746**
F11C-2A	9/18	1	HFW	34.80137	-78.98528	Lumbee	1	Filter	241	222	26	244	745	254	317	**746**
F22C-2B	9/18	1	HFW	35.046774	-78.366462	Cape Fear	1	Filter	241	222	26	244	745	254	317	**746**
F35C-A	9/28	1	HFW	34.34872	-78.40839	Waccam.	1	Suspension	241	222	26	244	745	254	317	**746**
F35C-B	9/28	1	HFW	34.34872	-78.40839	Waccam.	1	Suspension	241	222	26	244	745	254	317	**746**
F60C-1A	10/18	2	HFW	34.80137	-78.98528	Lumbee	1	Suspension	241	222	26	244	745	254	317	**746**
F84C-1A	10/18	2	HFW	35.377912	-77.822425	Neuse	1	Suspension	241	222	26	244	745	254	317	**746**
F5C-2A	9/18	1	HFW	35.313893	-77.945376	Neuse	2	Filter	241	252	26	243	750	38	34	**747**
F51C-A	9/28	1	HFW	35.380448	-77.86977	Neuse	1	Suspension	320	37	26	40	71	57	32	**749**
F10C-1A	9/18	1	HFW	35.463266	-77.669481	Neuse	3	Suspension	320	37	26	171	681	57	291	750

F55C-B	9/28	1	HFW	35.248663	-77.784804	Neuse	2	Suspension	320	37	26	171	681	57	291	750
F58C-2A	10/18	2	HFW	34.88732	-79.13168	Lumbee	1	Filter	320	37	26	171	681	57	291	750
F58C-2B	10/18	2	HFW	34.88732	-79.13168	Lumbee	1	Filter	320	37	26	171	681	57	291	750
F6C-1A	9/18	1	HFW	35.378341	-77.451253	Neuse	2	Suspension	320	37	26	171	681	57	291	750
F89C-1A	10/18	2	HFW	35.002593	-77.483371	Neuse	2	Suspension	320	37	26	171	681	57	291	750
F8C-1A	9/18	1	HFW	34.679601	-79.236498	Lumbee	2	Suspension	320	37	26	171	741	57	291	**751**
F2C-1A	9/18	1	HFW	34.823055	-79.057089	Lumbee	1	Suspension	367	33	26	171	375	5	32	**752**
F5C-1A	9/18	1	HFW	35.313893	-77.945376	Neuse	2	Suspension	368	15	26	48	638	74	86	**753**
F3C-2A	9/18	1	HFW	34.729631	-79.375567	Lumbee	1	Filter	369	33	26	171	749	411	79	**754**
F4C-2A	9/18	1	HFW	34.748189	-78.783604	Cape Fear	1	Filter	370	62	26	171	117	417	50	**755**
F4C-2B	9/18	1	HFW	34.748189	-78.783604	Cape Fear	1	Filter	370	62	26	171	117	417	50	**755**
F23C-2A	9/18	1	HFW	35.05744	-78.37756	Cape Fear	4	Filter	371	61	30	171	745	412	324	**756**
F28C-B	9/28	1	HFW	35.002593	-77.483371	Neuse	3	Suspension	372	37	26	144	638	57	79	**757**
F77C-2A	10/18	2	HFW	35.369768	-77.446194	Neuse	1	Filter	372	37	26	144	638	57	79	**757**
F77C-2B	10/18	2	HFW	35.369768	-77.446194	Neuse	1	Filter	372	37	26	144	638	57	79	**757**
F41C-A	9/28	1	HFW	34.52114	-79.17829	Lumbee	2	Suspension	373	33	26	247	746	403	325	**758**
F50C-A	9/28	1	HFW	35.377817	-77.822484	Neuse	1	Suspension	375	258	26	247	762	413	183	**760**
F50C-B	9/28	1	HFW	35.377817	-77.822484	Neuse	1	Suspension	375	258	26	247	762	413	183	**760**
F80C-1A	10/18	2	HFW	35.313954	-77.815157	Neuse	1	Suspension	375	258	26	247	762	413	183	**760**
F52C-A	9/28	1	HFW	35.313954	-77.815157	Neuse	1	Suspension	376	259	26	12	761	406	67	**761**
F52C-B	9/28	1	HFW	35.313954	-77.815157	Neuse	1	Suspension	376	259	26	12	761	406	67	**761**
F68C-1A	10/18	2	HFW	34.86359	-79.10484	Lumbee	1	Suspension	14	10	26	62	777	424	334	**807**
F83C-1A	10/18	2	HFW	35.05151	-78.37655	Cape Fear	N/A	Suspension	14	37	1	144	375	57	283	**808**
F68C-1B	10/18	2	HFW	34.86359	-79.10484	Lumbee	1	Suspension	14	37	228	241	745	405	222	**809**
F82C-1A	10/18	2	HFW	35.31346	-77.945928	Neuse	3	Suspension	55	49	26	250	123	414	32	**813**
F70C-1A	10/18	2	HFW	35.377912	-77.822425	Neuse	1	Suspension	59	158	26	40	766	419	56	**814**
F80C-2A	10/18	2	HFW	35.313954	-77.815157	Neuse	1	Filter	203	141	26	55	772	38	331	**817**
F86C-1A	10/18	2	HFW	35.1147	-78.4767	Cape Fear	1	Suspension	209	61	46	40	771	38	50	**818**
F61C-2A	10/18	2	HFW	34.624032	-79.012825	Lumbee	2	Filter	209	144	26	251	453	422	56	**819**
F96C-1A	10/19	2	HFW	34.368135	-78.460497	Waccam.	2	Suspension	216	141	26	48	638	246	317	**820**
F96C-1B	10/19	2	HFW	34.368135	-78.460497	Waccam.	2	Suspension	216	141	26	48	638	246	317	**820**
F87C-2A	10/18	2	HFW	35.05744	-78.37756	Cape Fear	3	Filter	368	15	186	48	638	74	86	**821**
F99C-2A	10/19	2	HFW	34.348935	-78.538171	Waccam.	2	Filter	368	15	186	48	638	74	86	**821**
F99C-2B	10/19	2	HFW	34.348935	-78.538171	Waccam.	2	Filter	368	15	186	48	638	74	86	**821**
F85C-1A	10/18	2	HFW	35.1506	-78.3819	Cape Fear	1	Suspension	376	151	46	55	770	38	50	**822**
F71C-1A	10/18	2	HFW	35.458092	-77.675781	Neuse	3	Suspension	378	141	26	48	169	43	324	**823**
F73C-1A	10/18	2	HFW	35.612725	-77.804619	Neuse	2	Suspension	379	260	26	144	169	420	32	**824**
F83C-1B	10/18	2	HFW	35.05151	-78.37655	Cape Fear	N/A	Suspension	379	260	26	144	169	420	32	**824**
F75C-1A	10/18	2	HFW	35.525188	-77.5227	Neuse	1	Suspension	380	62	26	240	767	97	329	**825**
F76C-1A	10/18	2	HFW	35.456645	-77.485555	Neuse	1	Suspension	381	33	26	241	117	417	50	**826**
F104C-2A	11/13	N/A	HFW	N/A	N/A	Lumbee	LB	Filter	381	158	227	248	109	418	79	**827**
F62C-1A	10/18	2	HFW	34.679601	-79.236498	Lumbee	2	Suspension	381	158	227	248	109	418	79	**827**
F82C-2A	10/18	2	HFW	35.31346	-77.945928	Neuse	3	Filter	382	15	186	48	638	74	86	**828**
F64C-1A	10/18	2	HFW	34.729651	-79.375575	Lumbee	1	Suspension	383	261	26	48	773	418	56	**829**
RBC101518-1-A	10/15	N/A	RBC	N/A	N/A	N/A	RBC	Filter	384	141	26	48	375	43	324	**830**
RBC101518-1-B	10/15	N/A	RBC	N/A	N/A	N/A	RBC	Filter	384	141	26	48	375	43	324	**830**
RBC101518-4-A	10/15	N/A	RBC	N/A	N/A	N/A	RBC	Filter	385	64	26	40	776	423	333	**831**
RBC101518-4-B	10/15	N/A	RBC	N/A	N/A	N/A	RBC	Filter	385	64	26	40	776	423	333	**831**

'Cluster C' *Arcobacter butzleri* and *Campylobacter jejuni* isolates

Isolate[a]	2018 Sample Date (mo/d)	Phase	Source[b]	Latitude	Longitude	Water-shed[d]	Water Body Type[e]	Enrichment Type	*aspA*	*atpA*	*glnA*	*gltA*	*glyA*	*pgm*	*tkt*	ST[f]
F72C-1A	10/18	2	HFW	35.379864	-77.450886	Neuse	3	Suspension	37	5	11	30	144	16	72	138
F20C-1A	9/18	1	HFW	35.377817	-77.822484	Neuse	2	Suspension	58	41	1	22	129	70	6	**740**
F97C-2A[g]	10/19	2	HFW	34.368135	-78.460497	Waccam.	1	Filter	200	224	6	61	176	40	180	**2866**
F97C-2B[g]	10/19	2	HFW	34.368135	-78.460497	Waccam.	1	Filter	200	224	6	61	176	40	180	**2866**

[a] Isolate designations starting with F are Hurricane Florence floodwater isolates; Isolate designations RBC are Rocky Branch Creek isolates, Raleigh, NC, USA. Designations after the hyphen indicate isolates derived from single colonies from the corresponding sample. For instance, F97C-2A and F97C-2B are two isolates, each derived from a single colony from enrichment of sample F97C.

[b] HFW: Hurricane Florence water sample; RBC: Rocky Branch Creek water sample.

[c] N/A: not available.

[d] Watersheds are as in Fig. 2 and included Lumbee, Neuse, Cape Fear, and Waccamaw (indicated Waccam.), a sub-basin of the Lumbee watershed.

[e] 1: Channel; 2: Floodplain; 3: Isolated ephemeral; 4: Other (large pond); LB: Lumbee Basin; RBC: Rocky Branch Creek.

[f] Novel sequence types in bold. *A. butzleri* and *C. jejuni* STs represent designations from different databases.

[g] *C. jejuni* isolates. *A. butzleri* and *C. jejuni* STs represent designations from different databases.

Supplementary Table 2: Source information and MLST data for strains used in Figure 4

Sequence types (STs) with > 1 strain in red; *** = not assigned to a clade in Figure 4; Unk. = unknown.

Clade	ST	Strain	Location	Date	Source (PubMLST)	Source (Figure 4)	*aspA*	*atpA*	*glnA*	*gltA*	*glyA*	*pgm*	*tkt*
**A**	**8**	RM5538	USA	Unk.	human stool	Human	3	2	20	30	160	107	82
**A**	**9**	14151	Thailand	2003	beef offal or meat	Ruminant	3	3	3	2	17	17	3
**A**	**10**	14109	France	2004	human stool	Human	3	3	3	3	2	3	3
**A**	**11**	14121	France	2003	human stool	Human	3	6	6	6	6	6	5
**A**	**12**	14159	Thailand	2003	chicken offal or meat	Poultry	3	17	16	20	25	21	7
**A**	**14**	RM5549	Thailand	Unk.	human stool	Human	3	24	2	45	166	120	84
**A**	**14**	RM5550	Thailand	Unk.	human stool	Human	3	24	2	45	166	120	84
**A**	**15**	RM5554	Thailand	Unk.	human stool	Human	3	32	3	34	168	44	36
**A**	**15**	14194	Vietnam	2002	human stool	Human	3	32	3	34	168	44	36
**A**	**16**	RM5230	Denmark	1997	human stool	Human	3	55	30	15	136	104	36
**A**	**16**	Ab-PV1	Spain	2015	other food	Other animal/food	3	55	30	15	136	104	36
**A**	**67**	14155	Thailand	2003	beef offal or meat	Ruminant	16	14	1	18	147	18	12
**A**	**68**	14183	Thailand	2003	eggs	Poultry	16	14	1	18	43	39	12
**A**	**110**	F79C-1A	USA	2018	environmental waters	Hurricane Florence Water	25	3	1	7	35	29	27
**A**	**110**	14170	Thailand	2003	chicken offal or meat	Poultry	25	3	1	7	35	29	27
**A**	**111**	14197	Vietnam	2002	human stool	Human	25	31	26	35	52	47	36
**A**	**111**	14208	Thailand	2005	human stool	Human	25	31	26	35	52	47	36
**A**	**112**	RM5530	USA	Unk.	human unspecified	Human	26	11	19	12	153	114	75
**A**	**125**	RM5545	Thailand	Unk.	human stool	Human	31	29	2	31	65	41	35
**A**	**125**	14190	Vietnam	2002	human stool	Human	31	29	2	31	65	41	35
**A**	**125**	RM5597	Unk.	Unk.	poultry carcass swab	Poultry	31	29	2	31	65	41	35
**A**	**126**	RM4465	USA	Unk.	human stool	Human	31	43	35	15	107	79	53
**A**	**127**	RM4468	USA	Unk.	human stool	Human	31	43	35	50	108	80	54
**A**	**128**	RM5520	Thailand	Unk.	human stool	Human	32	30	11	32	161	42	20
**A**	**128**	RM5539	Thailand	Unk.	human stool	Human	32	30	11	32	161	42	20
**A**	**128**	RM5553	Thailand	Unk.	human stool	Human	32	30	11	32	161	42	20
**A**	**128**	14191	Vietnam	2002	human stool	Human	32	30	11	32	161	42	20
**A**	**129**	RM5546	Thailand	Unk.	human stool	Human	33	31	27	33	22	43	4
**A**	**129**	RM5547	Thailand	Unk.	human stool	Human	33	31	27	33	22	43	4
**A**	**129**	RM5552	Thailand	Unk.	human stool	Human	33	31	27	33	22	43	4
**A**	**129**	14192	Vietnam	2002	human stool	Human	33	31	27	33	22	43	4
**A**	**130**	AF-ARCO-17	Thailand	Unk.	human	Human	34	12	2	34	58	46	38
**A**	**130**	AF-ARCO-88	Thailand	Unk.	human	Human	34	12	2	34	58	46	38
**A**	**130**	AF-ARCO-66	Thailand	Unk.	human	Human	34	12	2	34	58	46	38
**A**	**130**	14196	Vietnam	2002	human stool	Human	34	12	2	34	58	46	38

Clade	ST	Strain	Location	Date	Source (PubMLST)	Source (Figure 4)	*aspA*	*atpA*	*glnA*	*gltA*	*glyA*	*pgm*	*tkt*
**A**	**130**	14215	Thailand	2002	human stool	Human	34	12	2	34	58	46	38
**A**	**131**	14216	Thailand	2003	human stool	Human	35	11	2	35	59	47	36
**A**	**132**	RM4843	USA	Unk.	poultry carcass swab	Poultry	35	33	28	36	120	48	38
**A**	**133**	RM5556	Canada	Unk.	human stool	Human	35	33	28	36	170	48	38
**A**	**134**	14198	Vietnam	2002	human stool	Human	35	33	28	36	47	48	38
**A**	**135**	14221	Thailand	2003	chicken offal or meat	Poultry	35	33	19	20	62	48	38
**A**	**135**	14226	Thailand	2003	chicken offal or meat	Poultry	35	33	19	20	62	48	38
**A**	**136**	14234	Thailand	2003	chicken offal or meat	Poultry	35	33	28	37	76	48	38
**A**	**137**	14201	Thailand	2005	human stool	Human	36	3	29	37	87	22	4
**A**	**137**	14204	Thailand	2005	human stool	Human	36	3	29	37	87	22	4
**A**	**143**	RM5540	USA	Unk.	human stool	Human	38	3	24	63	162	59	45
**A**	**144**	14206	Thailand	2005	human stool	Human	38	35	26	7	51	51	4
**A**	**145**	RM5519	USA	Unk.	human stool	Human	39	5	1	34	107	109	38
**A**	**146**	RM5542	USA	Unk.	human stool	Human	39	5	2	39	164	109	38
**A**	**147**	14209	Thailand	2002	human stool	Human	39	7	17	2	53	52	41
**A**	**148**	14214	Thailand	2001	human stool	Human	39	33	2	39	57	56	38
**A**	**149**	14262	Thailand	2003	fish	Seafood	39	41	30	46	99	72	40
**A**	**150**	14263	Thailand	2003	squid	Seafood	39	42	27	47	100	73	20
**A**	**151**	14210	Thailand	2001	human stool	Human	40	36	3	38	54	53	38
**A**	**152**	14211	Thailand	2001	human stool	Human	41	11	30	12	55	54	42
**A**	**152**	14212	Thailand	2002	human stool	Human	41	11	30	12	55	54	42
**A**	**155**	14232	Thailand	2003	squid	Seafood	43	3	2	42	74	59	45
**A**	**165**	14249	Thailand	2003	shrimp	Seafood	49	3	29	37	87	66	4
**A**	**171**	RM4462	USA	Unk.	human stool	Human	54	14	33	15	105	77	4
**A**	**176**	RM4596	USA	Unk.	turkey	Poultry	57	46	37	22	115	16	31
**A**	**188**	RM5221	Turkey	2003	human stool	Human	67	13	2	58	87	99	68
**A**	**188**	RM5223	Turkey	2003	human stool	Human	67	13	2	58	87	99	68
**A**	**189**	RM5222	Turkey	2003	human stool	Human	68	54	2	59	131	100	69
**A**	**194**	RM5242	Sweden	1999	sheep	Ruminant	72	30	43	20	141	108	30
**A**	**197**	RM5525	Ireland	Unk.	pig	Pig	74	8	1	60	147	112	73
**A**	**197**	RM5526	Ireland	Unk.	bovine fetus, aborted	Ruminant	74	8	1	60	147	112	73
**A**	**202**	RM5541	USA	Unk.	human stool	Human	79	66	11	22	163	119	83
**A**	**204**	RM5559	USA	Unk.	human unspecified	Human	80	67	49	25	171	7	26
**A**	**292**	31166	UK	2007	cattle	Ruminant	3	124	113	7	348	199	62
**A**	**292**	31316	UK	2007	cattle	Ruminant	3	124	113	7	348	199	62
**A**	**292**	31318	UK	2007	cattle	Ruminant	3	124	113	7	348	199	62
**A**	**292**	31149	UK	2007	cattle	Ruminant	3	124	113	7	348	199	62

Clade	ST	Strain	Location	Date	Source (PubMLST)	Source (Figure 4)	*aspA*	*atpA*	*glnA*	*gltA*	*glyA*	*pgm*	*tkt*
**A**	**292**	31152	UK	2007	cattle	Ruminant	3	124	113	7	348	199	62
**A**	**292**	31169	UK	2007	cattle	Ruminant	3	124	113	7	348	199	62
**A**	**292**	31170	UK	2007	cattle	Ruminant	3	124	113	7	348	199	62
**A**	**292**	31155	UK	2007	cattle	Ruminant	3	124	113	7	348	199	62
**A**	**292**	31147	UK	2007	cattle	Ruminant	3	124	113	7	348	199	62
**A**	**292**	31150	UK	2007	cattle	Ruminant	3	124	113	7	348	199	62
**A**	**292**	31148	UK	2007	cattle	Ruminant	3	124	113	7	348	199	62
**A**	**300**	30642	UK	2007	cattle	Ruminant	35	33	28	36	207	48	38
**A**	**300**	30645	UK	2007	cattle	Ruminant	35	33	28	36	207	48	38
**A**	**302**	34720	UK	2008	cattle	Ruminant	38	3	2	63	364	59	45
**A**	**302**	35177	UK	2008	cattle	Ruminant	38	3	2	63	364	59	45
**A**	**302**	35178	UK	2008	cattle	Ruminant	38	3	2	63	364	59	45
**A**	**302**	35228	UK	2008	cattle	Ruminant	38	3	2	63	364	59	45
**A**	**302**	35235	UK	2008	cattle	Ruminant	38	3	2	63	364	59	45
**A**	**307**	31171	UK	2007	cattle	Ruminant	160	125	26	60	349	200	73
**A**	**307**	31173	UK	2007	cattle	Ruminant	160	125	26	60	349	200	73
**A**	**307**	31174	UK	2007	cattle	Ruminant	160	125	26	60	349	200	73
**A**	**309**	34029	UK	2008	cattle	Ruminant	170	66	26	15	154	215	162
**A**	**309**	34030	UK	2008	cattle	Ruminant	170	66	26	15	154	215	162
**A**	**309**	34043	UK	2008	cattle	Ruminant	170	66	26	15	154	215	162
**A**	**312**	6	Belgium	2007	pig	Pig	39	33	2	129	164	109	4
**A**	**312**	28	Belgium	2007	pig	Pig	39	33	2	129	164	109	4
**A**	**312**	59	Belgium	2007	pig	Pig	39	33	2	129	164	109	4
**A**	**312**	118	Belgium	2007	pig	Pig	39	33	2	129	164	109	4
**A**	**312**	131	Belgium	2007	pig	Pig	39	33	2	129	164	109	4
**A**	**312**	2b	Belgium	2007	pig	Pig	39	33	2	129	164	109	4
**A**	**322**	B30	Belgium	2007	pig	Pig	15	128	118	132	381	225	166
**A**	**327**	35681	UK	2008	cattle	Ruminant	3	134	113	2	412	199	62
**A**	**345**	35296	UK	2008	cattle	Ruminant	43	41	1	19	358	20	170
**A**	**346**	34597	UK	2008	cattle	Ruminant	43	41	11	17	372	16	161
**A**	**346**	35292	UK	2008	cattle	Ruminant	43	41	11	17	372	16	161
**A**	**346**	35293	UK	2008	cattle	Ruminant	43	41	11	17	372	16	161
**A**	**346**	35294	UK	2008	cattle	Ruminant	43	41	11	17	372	16	161
**A**	**346**	35295	UK	2008	cattle	Ruminant	43	41	11	17	372	16	161
**A**	**357**	36076	UK	2008	cattle	Ruminant	183	135	1	12	417	102	160
**A**	**357**	36088	UK	2008	cattle	Ruminant	183	135	1	12	417	102	160
**A**	**357**	36074	UK	2008	cattle	Ruminant	183	135	1	12	417	102	160

Clade	ST	Strain	Location	Date	Source (PubMLST)	Source (Figure 4)	*aspA*	*atpA*	*glnA*	*gltA*	*glyA*	*pgm*	*tkt*
**A**	**370**	03.01.13	Denmark	2011	chicken	Poultry	206	143	2	37	166	53	162
**A**	**370**	03.01.19.01	Denmark	2011	chicken	Poultry	206	143	2	37	166	53	162
**A**	**370**	03.01.19.02	Denmark	2011	chicken	Poultry	206	143	2	37	166	53	162
**A**	**377**	AB-P1	Spain	2011	chicken offal or meat	Poultry	208	2	26	142	455	39	19
**A**	**461**	AB-FW42	Spain	2010	environmental waters	Environmental waters	3	30	16	23	516	83	31
**A**	**471**	AB-FW25	Spain	2010	environmental waters	Environmental waters	213	140	2	129	499	109	4
**A**	**481**	AB-FW63	Spain	2010	environmental waters	Environmental waters	72	30	43	20	515	108	30
**A**	**485**	1966730	Spain	2014	human stool	Human	80	67	49	12	47	44	33
**A**	**492**	HJXXIII-6	Spain	2014	human stool	Human	80	67	49	25	523	266	9
**A**	**494**	HSJR-4	Spain	2013	human stool	Human	80	67	49	12	524	2	33
**A**	**495**	HSJR-5	Spain	2014	human stool	Human	80	67	49	25	171	265	26
**A**	**496**	HSJR-6	Spain	2015	human stool	Human	38	30	11	20	518	264	208
**A**	**500**	Ab-RW5	Spain	2010	environmental waters	Environmental waters	57	168	43	2	6	17	4
**A**	**515**	Ab-PV9	Spain	2015	other food	Other animal/food	57	170	19	20	542	277	221
**A**	**531**	Ab-Z3	Spain	2015	other food	Other animal/food	3	5	11	30	550	20	4
**A**	**537**	6V	Italy	2015	clam	Seafood	236	161	1	183	521	296	207
**A**	**582**	AF-ARCO-19	Thailand	Unk.	human stool	Human	38	35	26	20	165	51	4
**A**	**583**	AF-ARCO-65	Thailand	Unk.	human stool	Human	25	194	127	32	52	42	20
**A**	**585**	AF-ARCO-59	Thailand	Unk.	human stool	Human	173	41	19	6	599	77	263
**A**	**586**	Ab-CN2	Spain	2015	other food	Other animal/food	256	17	151	15	552	288	12
**A**	**589**	Ab-Z5	Spain	2015	other food	Other animal/food	257	185	152	37	421	287	19
**A**	**592**	AF-ARCO-67	Thailand	Unk.	human stool	Human	39	196	180	37	73	322	264
**A**	**613**	AF-ARCO-63	Thailand	Unk.	human stool	Human	285	66	1	20	601	326	261
**A**	**615**	AF-ARCO-74	Thailand	Unk.	human stool	Human	40	17	2	63	54	325	31
**A**	**616**	AF-ARCO-58	Thailand	Unk.	human stool	Human	3	3	30	15	598	330	4
**A**	**617**	AF-ARCO-60	Thailand	Unk.	human stool	Human	80	67	49	30	524	263	267
**A**	**618**	AF-ARCO-64	Thailand	Unk.	human stool	Human	292	32	152	34	46	327	36
**A**	**619**	AF-ARCO-68	Thailand	Unk.	human stool	Human	293	42	3	20	596	331	4
**A**	**621**	AF-ARCO-72	Thailand	Unk.	human stool	Human	3	31	1	20	545	326	261
**A**	**639**	HJXXIII-12	Spain	2016	human stool	Human	80	67	49	25	171	290	26
**A**	**642**	HSJR-7	Spain	2016	human stool	Human	39	33	2	128	595	317	4
**A**	**643**	HJXXIII-15	Spain	2016	human stool	Human	80	67	49	12	524	321	33
**A**	**653**	39/O	Italy	2015	other food	Other animal/food	3	42	2	15	684	21	4
**A**	**679**	HJXXIII-16	Spain	2016	human stool	Human	80	67	49	23	524	30	23
**A**	**718**	F9C-1A	USA	2018	environmental waters	Hurricane Florence Water	3	41	11	15	742	410	4
**A**	**719**	F49C-A	USA	2018	environmental waters	Hurricane Florence Water	3	41	11	20	54	70	31
**A**	**720**	F48C-A	USA	2018	environmental waters	Hurricane Florence Water	3	42	11	12	759	338	4

Clade	ST	Strain	Location	Date	Source (PubMLST)	Source (Figure 4)	*aspA*	*atpA*	*glnA*	*gltA*	*glyA*	*pgm*	*tkt*
**A**	**720**	F48C-B	USA	2018	environmental waters	Hurricane Florence Water	3	42	11	12	759	338	4
**A**	**721**	F11C-1A	USA	2018	environmental waters	Hurricane Florence Water	3	42	181	15	143	407	320
**A**	**721**	F69C-1A	USA	2018	environmental waters	Hurricane Florence Water	3	42	181	15	143	407	320
**A**	**722**	F22C-1A	USA	2018	environmental waters	Hurricane Florence Water	3	251	2	242	748	90	264
**A**	**735**	F27C-B	USA	2018	environmental waters	Hurricane Florence Water	25	30	1	246	35	29	27
**A**	**736**	F20C-2B	USA	2018	environmental waters	Hurricane Florence Water	38	14	1	2	599	17	323
**A**	**736**	F65C-1A	USA	2018	environmental waters	Hurricane Florence Water	38	14	1	2	599	17	323
**A**	**737**	F9C-2A	USA	2018	environmental waters	Hurricane Florence Water	39	33	2	129	595	109	4
**A**	**738**	F6C-2A	USA	2018	environmental waters	Hurricane Florence Water	40	66	113	20	751	101	4
**A**	**739**	F39C-B	USA	2018	environmental waters	Hurricane Florence Water	57	14	26	12	756	408	19
**A**	**748**	F17C-2B	USA	2018	environmental waters	Hurricane Florence Water	292	30	127	239	754	48	20
**A**	**759**	F45C-B	USA	2018	environmental waters	Hurricane Florence Water	374	257	225	44	757	415	326
**A**	**762**	F53C-A	USA	2018	environmental waters	Hurricane Florence Water	377	41	226	22	765	238	328
**A**	**762**	F53C-B	USA	2018	environmental waters	Hurricane Florence Water	377	41	226	22	765	238	328
**A**	**806**	F59C-1A	USA	2018	environmental waters	Hurricane Florence Water	3	30	1	15	769	421	27
**A**	**810**	F78C-1A	USA	2018	environmental waters	Hurricane Florence Water	31	42	11	15	768	338	330
**A**	**811**	F84C-1B	USA	2018	environmental waters	Hurricane Florence Water	39	3	2	34	74	35	200
**A**	**812**	RBC101518-3-A	USA	2018	environmental waters	RBC creek water	39	5	1	34	775	109	38
**A**	**812**	RBC101518-3-B	USA	2018	environmental waters	RBC creek water	39	5	1	34	775	109	38
**A**	**815**	F104C-1A	USA	2018	environmental waters	Hurricane Florence Water	181	3	2	30	404	227	332
**A**	**816**	RBC101518-2-A	USA	2018	environmental waters	RBC creek water	183	262	2	2	774	56	20
**A**	**816**	RBC101518-2-B	USA	2018	environmental waters	RBC creek water	183	262	2	2	774	56	20
**B**	**59**	14148	Thailand	2003	beef offal or meat	Ruminant	14	13	14	16	16	15	15
**B**	**60**	RM4473	USA	Unk.	human stool	Human	14	17	35	51	111	82	57
**B**	**61**	14180	Thailand	2003	pork offal or meat	Pig	14	27	15	28	40	36	32
**B**	**64**	RM5535	USA	Unk.	human unspecified	Human	14	63	26	62	156	118	79
**B**	**65**	RM5562	USA	Unk.	human stool	Human	14	68	50	40	174	122	79
**B**	**69**	14156	Thailand	2003	beef offal or meat	Ruminant	17	15	15	12	23	19	17
**B**	**69**	14157	Thailand	2003	beef offal or meat	Ruminant	17	15	15	12	23	19	17
**B**	**70**	RM5533	USA	Unk.	human blood culture	Human	17	62	46	55	24	116	78
**B**	**71**	RM5555	Canada	Unk.	human stool	Human	17	62	26	53	169	121	79
**B**	**94**	14199	Thailand	2004	human stool	Human	21	22	21	24	48	27	25
**B**	**94**	14259	Thailand	2003	pork offal or meat	Pig	21	22	21	24	48	27	25
**B**	**94**	14168	Thailand	2003	chicken offal or meat	Poultry	21	22	21	24	48	27	25
**B**	**94**	AF-ARCO-79	Thailand	Unk.	chicken offal or meat	Poultry	21	22	21	24	48	27	25
**B**	**118**	14182	Thailand	2003	pork offal or meat	Pig	29	28	26	29	42	38	34
**B**	**142**	14229	Thailand	2003	chicken offal or meat	Poultry	37	37	32	40	71	57	32

Clade	ST	Strain	Location	Date	Source (PubMLST)	Source (Figure 4)	*aspA*	*atpA*	*glnA*	*gltA*	*glyA*	*pgm*	*tkt*
**B**	**170**	RM1588	USA	Unk.	chicken offal or meat	Poultry	53	15	15	48	101	74	50
**B**	**173**	RM5531	USA	Unk.	human stool	Human	55	60	26	40	154	79	76
**B**	**178**	RM4835	USA	Unk.	turkey	Poultry	59	47	26	53	117	88	60
**B**	**179**	Ab-RW14	Spain	2010	environmental waters	Environmental waters	60	15	15	54	183	89	61
**B**	**179**	RM5598	Unk.	Unk.	poultry carcass swab	Poultry	60	15	15	54	183	89	61
**B**	**179**	RM4837	USA	Unk.	turkey	Poultry	60	15	15	54	183	89	61
**B**	**180**	RM4846	USA	Unk.	pork offal or meat	Pig	60	15	15	54	122	89	61
**B**	**180**	RM4847	USA	Unk.	pork offal or meat	Pig	60	15	15	54	122	89	61
**B**	**181**	RM4841	USA	Unk.	poultry carcass swab	Poultry	61	48	21	24	119	27	25
**B**	**181**	RM4842	USA	Unk.	poultry carcass swab	Poultry	61	48	21	24	119	27	25
**B**	**184**	RM5211	Nigeria	2000	broiler environment	Poultry	63	51	21	24	126	27	25
**B**	**186**	F44C-A	USA	2018	environmental waters	Hurricane Florence Water	65	53	26	57	137	97	67
**B**	**186**	F44C-B	USA	2018	environmental waters	Hurricane Florence Water	65	53	26	57	137	97	67
**B**	**186**	RM5217	Nigeria	2000	broiler environment	Poultry	65	53	26	57	137	97	67
**B**	**199**	RM5532	USA	Unk.	human stool	Human	76	61	26	61	155	115	77
**B**	**201**	RM5536	USA	Unk.	human stool	Human	78	64	47	48	157	97	80
**B**	**205**	RM5594	Unk.	Unk.	pig	Pig	81	69	26	66	182	124	86
**B**	**314**	F69C-2A	USA	2018	environmental waters	Hurricane Florence Water	14	15	26	131	375	223	34
**B**	**314**	A39	Belgium	2007	pig	Pig	14	15	26	131	375	223	34
**B**	**314**	A42	Belgium	2007	pig	Pig	14	15	26	131	375	223	34
**B**	**314**	A43	Belgium	2007	pig	Pig	14	15	26	131	375	223	34
**B**	**314**	A45	Belgium	2007	pig	Pig	14	15	26	131	375	223	34
**B**	**314**	A62	Belgium	2007	pig	Pig	14	15	26	131	375	223	34
**B**	**314**	A63	Belgium	2007	pig	Pig	14	15	26	131	375	223	34
**B**	**314**	A65b	Belgium	2007	pig	Pig	14	15	26	131	375	223	34
**B**	**314**	A66	Belgium	2007	pig	Pig	14	15	26	131	375	223	34
**B**	**358**	Ab-A6	Spain	2011	clam	Seafood	203	140	26	140	385	38	86
**B**	**371**	03.02.01.02	Denmark	2011	chicken	Poultry	14	49	26	55	449	102	176
**B**	**371**	03.02.04	Denmark	2011	chicken	Poultry	14	49	26	55	449	102	176
**B**	**373**	01.02.01	Denmark	2011	chicken	Poultry	205	144	123	55	447	233	177
**B**	**373**	01.02.04	Denmark	2011	chicken	Poultry	205	144	123	55	447	233	177
**B**	**373**	01.02.05	Denmark	2011	chicken	Poultry	205	144	123	55	447	233	177
**B**	**373**	01.02.07.02	Denmark	2011	chicken	Poultry	205	144	123	55	447	233	177
**B**	**373**	03.02.05	Denmark	2011	chicken	Poultry	205	144	123	55	447	233	177
**B**	**378**	AB-P2	Spain	2011	chicken offal or meat	Poultry	209	146	26	143	453	237	182
**B**	**378**	Ab-P3	Spain	2011	chicken offal or meat	Poultry	209	146	26	143	453	237	182
**B**	**383**	AB-P14	Spain	2011	chicken offal or meat	Poultry	203	24	126	145	452	235	181

Clade	ST	Strain	Location	Date	Source (PubMLST)	Source (Figure 4)	*aspA*	*atpA*	*glnA*	*gltA*	*glyA*	*pgm*	*tkt*
**B**	**396**	Ab-A1	Spain	2011	clam	Seafood	14	151	26	55	466	241	56
**B**	**403**	Ab-M1	Spain	2011	mussel	Seafood	212	149	26	144	462	38	183
**B**	**404**	Ab-A2	Spain	2011	clam	Seafood	212	149	26	144	469	38	183
**B**	**404**	Ab-A3	Spain	2011	clam	Seafood	212	149	26	144	469	38	183
**B**	**405**	Ab-M4	Spain	2011	mussel	Seafood	213	140	26	140	120	242	86
**B**	**406**	Ab-RW9	Spain	2010	environmental waters	Environmental waters	214	62	128	148	465	244	186
**B**	**406**	Ab-CN1	Spain	2015	other food	Other animal/food	214	62	128	148	465	244	186
**B**	**406**	Ab-A11	Spain	2011	clam	Seafood	214	62	128	148	465	244	186
**B**	**406**	Ab-A9	Spain	2011	clam	Seafood	214	62	128	148	465	244	186
**B**	**406**	1V	Italy	2015	clam	Seafood	214	62	128	148	465	244	186
**B**	**407**	Ab-A8	Spain	2011	clam	Seafood	216	62	26	144	474	246	188
**B**	**460**	F21C-1B	USA	2018	environmental waters	Hurricane Florence Water	234	15	26	164	375	260	176
**B**	**460**	F23C-1A	USA	2018	environmental waters	Hurricane Florence Water	234	15	26	164	375	260	176
**B**	**460**	F7C-2A	USA	2018	environmental waters	Hurricane Florence Water	234	15	26	164	375	260	176
**B**	**464**	AB-FW10	Spain	2010	environmental waters	Environmental waters	39	33	26	140	457	38	50
**B**	**474**	AB-FW28	Spain	2010	environmental waters	Environmental waters	81	62	128	148	119	244	183
**B**	**474**	F8C-2B	USA	2018	environmental waters	Hurricane Florence Water	81	62	128	148	119	244	183
**B**	**499**	Ab-RW4	Spain	2010	environmental waters	Environmental waters	246	141	26	37	536	38	79
**B**	**501**	Ab-RW6	Spain	2010	environmental waters	Environmental waters	59	169	147	55	537	273	213
**B**	**502**	Ab-RW7	Spain	2010	environmental waters	Environmental waters	60	15	15	55	538	89	61
**B**	**511**	Ab-CZ7	Spain	2015	other food	Other animal/food	63	51	21	24	526	27	25
**B**	**511**	Ab-CZ8	Spain	2015	other food	Other animal/food	63	51	21	24	526	27	25
**B**	**523**	Ab-PV5	Spain	2015	other food	Other animal/food	241	61	30	171	375	276	220
**B**	**527**	Ab-PV2	Spain	2015	other food	Other animal/food	63	51	21	24	529	27	25
**B**	**530**	Ab-BER5	Spain	2015	other food	Other animal/food	14	53	128	170	205	97	228
**B**	**591**	AF-ARCO-90	Thailand	Unk.	human stool	Human	291	195	173	198	602	318	222
**B**	**614**	AF-ARCO-73	Thailand	Unk.	human stool	Human	81	62	26	144	597	328	260
**B**	**649**	F76C-2A	USA	2018	environmental waters	Hurricane Florence Water	14	37	205	144	109	57	283
**B**	**651**	34/O	Italy	2015	other food	Other animal/food	268	186	153	123	635	306	210
**B**	**652**	26/O	Italy	2015	other food	Other animal/food	81	62	128	148	465	244	183
**B**	**723**	F49C-B	USA	2018	environmental waters	Hurricane Florence Water	5	33	26	62	761	416	327
**B**	**724**	F12C-2B	USA	2018	environmental waters	Hurricane Florence Water	5	62	224	55	753	409	204
**B**	**725**	F45C-A	USA	2018	environmental waters	Hurricane Florence Water	5	158	128	248	109	418	79
**B**	**726**	F13C-1A	USA	2018	environmental waters	Hurricane Florence Water	14	37	26	241	745	405	222
**B**	**726**	F39C-A	USA	2018	environmental waters	Hurricane Florence Water	14	37	26	241	745	405	222
**B**	**727**	F51C-B	USA	2018	environmental waters	Hurricane Florence Water	14	49	26	55	763	414	32
**B**	**728**	F1C-1A	USA	2018	environmental waters	Hurricane Florence Water	14	62	26	240	738	406	317

Clade	ST	Strain	Location	Date	Source (PubMLST)	Source (Figure 4)	*aspA*	*atpA*	*glnA*	*gltA*	*glyA*	*pgm*	*tkt*
**B**	**728**	F1C-1B	USA	2018	environmental waters	Hurricane Florence Water	14	62	26	240	738	406	317
**B**	**729**	F3C-1A	USA	2018	environmental waters	Hurricane Florence Water	14	151	222	144	739	409	76
**B**	**729**	F18C-1A	USA	2018	environmental waters	Hurricane Florence Water	14	151	222	144	739	409	76
**B**	**730**	F18C-2A	USA	2018	environmental waters	Hurricane Florence Water	14	253	26	144	755	223	322
**B**	**730**	F21C-2A	USA	2018	environmental waters	Hurricane Florence Water	14	253	26	144	755	223	322
**B**	**730**	F87C-1A	USA	2018	environmental waters	Hurricane Florence Water	14	253	26	144	755	223	322
**B**	**731**	F27C-A	USA	2018	environmental waters	Hurricane Florence Water	14	254	26	245	42	404	79
**B**	**732**	F28C-A	USA	2018	environmental waters	Hurricane Florence Water	14	255	50	40	174	122	79
**B**	**733**	F43C-A	USA	2018	environmental waters	Hurricane Florence Water	14	256	26	131	375	223	34
**B**	**733**	F43C-B	USA	2018	environmental waters	Hurricane Florence Water	14	256	26	131	375	223	34
**B**	**734**	F17C-1A	USA	2018	environmental waters	Hurricane Florence Water	17	62	223	40	746	43	321
**B**	**734**	F55C-A	USA	2018	environmental waters	Hurricane Florence Water	17	62	223	40	746	43	321
**B**	**734**	F71C-2A	USA	2018	environmental waters	Hurricane Florence Water	17	62	223	40	746	43	321
**B**	**741**	F47C-A	USA	2018	environmental waters	Hurricane Florence Water	65	53	26	249	638	97	67
**B**	**741**	F47C-B	USA	2018	environmental waters	Hurricane Florence Water	65	53	26	249	638	97	67
**B**	**742**	F2C-2A	USA	2018	environmental waters	Hurricane Florence Water	209	61	26	171	375	254	321
**B**	**743**	F7C-1A	USA	2018	environmental waters	Hurricane Florence Water	212	149	26	144	453	38	183
**B**	**744**	F14C-1A	USA	2018	environmental waters	Hurricane Florence Water	212	149	26	144	469	38	222
**B**	**745**	F14C-2A	USA	2018	environmental waters	Hurricane Florence Water	241	33	26	53	117	254	317
**B**	**746**	F11C-2A	USA	2018	environmental waters	Hurricane Florence Water	241	222	26	244	745	254	317
**B**	**746**	F22C-2B	USA	2018	environmental waters	Hurricane Florence Water	241	222	26	244	745	254	317
**B**	**746**	F35C-A	USA	2018	environmental waters	Hurricane Florence Water	241	222	26	244	745	254	317
**B**	**746**	F35C-B	USA	2018	environmental waters	Hurricane Florence Water	241	222	26	244	745	254	317
**B**	**746**	F60C-1A	USA	2018	environmental waters	Hurricane Florence Water	241	222	26	244	745	254	317
**B**	**746**	F84C-1A	USA	2018	environmental waters	Hurricane Florence Water	241	222	26	244	745	254	317
**B**	**746**	F105C-2A	USA	2018	environmental waters	Hurricane Florence Water	241	222	26	244	745	254	317
**B**	**747**	F5C-2A	USA	2018	environmental waters	Hurricane Florence Water	241	252	26	243	750	38	34
**B**	**749**	F51C-A	USA	2018	environmental waters	Hurricane Florence Water	320	37	26	40	71	57	32
**B**	**750**	F6C-1A	USA	2018	environmental waters	Hurricane Florence Water	320	37	26	171	681	57	291
**B**	**750**	F10C-1A	USA	2018	environmental waters	Hurricane Florence Water	320	37	26	171	681	57	291
**B**	**750**	F55C-B	USA	2018	environmental waters	Hurricane Florence Water	320	37	26	171	681	57	291
**B**	**750**	F89C-1A	USA	2018	environmental waters	Hurricane Florence Water	320	37	26	171	681	57	291
**B**	**750**	F58C-2A	USA	2018	environmental waters	Hurricane Florence Water	320	37	26	171	681	57	291
**B**	**750**	F58C-2B	USA	2018	environmental waters	Hurricane Florence Water	320	37	26	171	681	57	291
**B**	**750**	CT83	Nigeria	2016	cattle	Ruminant	320	37	26	171	681	57	291
**B**	**751**	F8C-1A	USA	2018	environmental waters	Hurricane Florence Water	320	37	26	171	741	57	291
**B**	**752**	F2C-1A	USA	2018	environmental waters	Hurricane Florence Water	367	33	26	171	375	5	32

Clade	ST	Strain	Location	Date	Source (PubMLST)	Source (Figure 4)	*aspA*	*atpA*	*glnA*	*gltA*	*glyA*	*pgm*	*tkt*
**B**	**753**	F5C-1A	USA	2018	environmental waters	Hurricane Florence Water	368	15	26	48	638	74	86
**B**	**754**	F3C-2A	USA	2018	environmental waters	Hurricane Florence Water	369	33	26	171	749	411	79
**B**	**755**	F4C-2A	USA	2018	environmental waters	Hurricane Florence Water	370	62	26	171	117	417	50
**B**	**755**	F4C-2B	USA	2018	environmental waters	Hurricane Florence Water	370	62	26	171	117	417	50
**B**	**756**	F23C-2A	USA	2018	environmental waters	Hurricane Florence Water	371	61	30	171	745	412	324
**B**	**757**	F28C-B	USA	2018	environmental waters	Hurricane Florence Water	372	37	26	144	638	57	79
**B**	**757**	F77C-2A	USA	2018	environmental waters	Hurricane Florence Water	372	37	26	144	638	57	79
**B**	**757**	F77C-2B	USA	2018	environmental waters	Hurricane Florence Water	372	37	26	144	638	57	79
**B**	**758**	F41C-A	USA	2018	environmental waters	Hurricane Florence Water	373	33	26	247	746	403	325
**B**	**760**	F50C-A	USA	2018	environmental waters	Hurricane Florence Water	375	258	26	247	762	413	183
**B**	**760**	F50C-B	USA	2018	environmental waters	Hurricane Florence Water	375	258	26	247	762	413	183
**B**	**760**	F80C-1A	USA	2018	environmental waters	Hurricane Florence Water	375	258	26	247	762	413	183
**B**	**761**	F52C-A	USA	2018	environmental waters	Hurricane Florence Water	376	259	26	12	761	406	67
**B**	**761**	F52C-B	USA	2018	environmental waters	Hurricane Florence Water	376	259	26	12	761	406	67
**B**	**807**	F68C-1A	USA	2018	environmental waters	Hurricane Florence Water	14	10	26	62	777	424	334
**B**	**808**	F83C-1A	USA	2018	environmental waters	Hurricane Florence Water	14	37	1	144	375	57	283
**B**	**809**	F68C-1B	USA	2018	environmental waters	Hurricane Florence Water	14	37	228	241	745	405	222
**B**	**813**	F82C-1A	USA	2018	environmental waters	Hurricane Florence Water	55	49	26	250	123	414	32
**B**	**814**	F70C-1A	USA	2018	environmental waters	Hurricane Florence Water	59	158	26	40	766	419	56
**B**	**817**	F80C-2A	USA	2018	environmental waters	Hurricane Florence Water	203	141	26	55	772	38	331
**B**	**818**	F86C-1A	USA	2018	environmental waters	Hurricane Florence Water	209	61	46	40	771	38	50
**B**	**819**	F61C-2A	USA	2018	environmental waters	Hurricane Florence Water	209	144	26	251	453	422	56
**B**	**820**	F96C-1A	USA	2018	environmental waters	Hurricane Florence Water	216	141	26	48	638	246	317
**B**	**820**	F96C-1B	USA	2018	environmental waters	Hurricane Florence Water	216	141	26	48	638	246	317
**B**	**821**	F87C-2A	USA	2018	environmental waters	Hurricane Florence Water	368	15	186	48	638	74	86
**B**	**821**	F99C-2A	USA	2018	environmental waters	Hurricane Florence Water	368	15	186	48	638	74	86
**B**	**821**	F99C-2B	USA	2018	environmental waters	Hurricane Florence Water	368	15	186	48	638	74	86
**B**	**822**	F85C-1A	USA	2018	environmental waters	Hurricane Florence Water	376	151	46	55	770	38	50
**B**	**823**	F71C-1A	USA	2018	environmental waters	Hurricane Florence Water	378	141	26	48	169	43	324
**B**	**824**	F73C-1A	USA	2018	environmental waters	Hurricane Florence Water	379	260	26	144	169	420	32
**B**	**824**	F83C-1B	USA	2018	environmental waters	Hurricane Florence Water	379	260	26	144	169	420	32
**B**	**825**	F75C-1A	USA	2018	environmental waters	Hurricane Florence Water	380	62	26	240	767	97	329
**B**	**826**	F76C-1A	USA	2018	environmental waters	Hurricane Florence Water	381	33	26	241	117	417	50
**B**	**827**	F62C-1A	USA	2018	environmental waters	Hurricane Florence Water	381	158	227	248	109	418	79
**B**	**827**	F104C-2A	USA	2018	environmental waters	Hurricane Florence Water	381	158	227	248	109	418	79
**B**	**828**	F82C-2A	USA	2018	environmental waters	Hurricane Florence Water	382	15	186	48	638	74	86
**B**	**829**	F64C-1A	USA	2018	environmental waters	Hurricane Florence Water	383	261	26	48	773	418	56

Clade	ST	Strain	Location	Date	Source (PubMLST)	Source (Figure 4)	*aspA*	*atpA*	*glnA*	*gltA*	*glyA*	*pgm*	*tkt*
**B**	**830**	RBC101518-1-A	USA	2018	environmental waters	RBC creek water	384	141	26	48	375	43	324
**B**	**830**	RBC101518-1-B	USA	2018	environmental waters	RBC creek water	384	141	26	48	375	43	324
**B**	**831**	RBC101518-4-A	USA	2018	environmental waters	RBC creek water	385	64	26	40	776	423	333
**B**	**831**	RBC101518-4-B	USA	2018	environmental waters	RBC creek water	385	64	26	40	776	423	333
**C**	**7**	RM5529	USA	Unk.	human stool	Human	2	59	45	15	151	113	2
**C**	**13**	14224	Thailand	2003	chicken offal or meat	Poultry	3	17	17	20	67	53	44
**C**	**21**	RM4844	USA	Unk.	pork offal or meat	Pig	5	5	5	19	121	86	10
**C**	**21**	RM4845	USA	Unk.	pork offal or meat	Pig	5	5	5	19	121	86	10
**C**	**23**	RM5566	Unk.	Unk.	pig	Pig	5	5	5	5	179	11	4
**C**	**24**	RM5592	Unk.	Unk.	pig	Pig	5	5	9	5	8	50	85
**C**	**24**	RM5593	Unk.	Unk.	pig	Pig	5	5	9	5	8	50	85
**C**	**24**	RM5595	Unk.	Unk.	pig	Pig	5	5	9	5	8	50	85
**C**	**26**	14118	France	2003	human stool	Human	5	5	5	5	4	5	4
**C**	**26**	14119	France	2003	human stool	Human	5	5	5	5	4	5	4
**C**	**26**	14120	France	2003	human stool	Human	5	5	5	5	4	5	4
**C**	**35**	14231	Thailand	2003	pork offal or meat	Pig	6	2	2	19	73	58	20
**C**	**36**	RM4849	USA	Unk.	pork offal or meat	Pig	6	5	38	7	144	93	13
**C**	**37**	14122	France	2003	human stool	Human	6	5	7	7	7	7	6
**C**	**46**	14123	France	2003	human stool	Human	7	7	7	7	186	8	7
**C**	**48**	14125	France	2003	human stool	Human	9	5	9	9	120	10	9
**C**	**52**	RM4591	USA	Unk.	turkey	Poultry	11	2	11	44	56	84	40
**C**	**54**	14145	Thailand	2003	beef offal or meat	Ruminant	11	10	11	13	13	14	13
**C**	**56**	14220	Thailand	2003	pork offal or meat	Pig	12	11	2	14	14	8	14
**C**	**56**	14146	Thailand	2003	beef offal or meat	Ruminant	12	11	2	14	14	8	14
**C**	**56**	14150	Thailand	2003	beef offal or meat	Ruminant	12	11	2	14	14	8	14
**C**	**56**	14153	Thailand	2003	beef offal or meat	Ruminant	12	11	2	14	14	8	14
**C**	**57**	14149	Thailand	2003	beef offal or meat	Ruminant	12	11	2	9	61	16	16
**C**	**58**	14147	Thailand	2003	beef offal or meat	Ruminant	13	12	13	15	15	8	6
**C**	**58**	14154	Thailand	2003	beef offal or meat	Ruminant	13	12	13	15	15	8	6
**C**	**63**	RM5132	USA	2005	chicken offal or meat	Poultry	14	50	30	25	152	95	64
**C**	**66**	14152	Thailand	2003	beef offal or meat	Ruminant	15	10	1	17	19	2	13
**C**	**72**	14158	Thailand	2003	chicken offal or meat	Poultry	18	16	11	19	24	20	18
**C**	**72**	14164	Thailand	2003	chicken offal or meat	Poultry	18	16	11	19	24	20	18
**C**	**73**	RM5214	Nigeria	2000	broiler environment	Poultry	19	17	17	12	67	77	65
**C**	**74**	14160	Thailand	2003	chicken offal or meat	Poultry	19	17	17	20	26	22	19
**C**	**74**	AF-ARCO-75	Thailand	Unk.	chicken offal or meat	Poultry	19	17	17	20	26	22	19
**C**	**75**	RM5521	USA	Unk.	human stool	Human	20	2	2	30	143	110	71

Clade	ST	Strain	Location	Date	Source (PubMLST)	Source (Figure 4)	*aspA*	*atpA*	*glnA*	*gltA*	*glyA*	*pgm*	*tkt*
**C**	**76**	14213	Thailand	2001	human stool	Human	20	2	11	22	56	55	40
**C**	**77**	14257	Thailand	2003	chicken offal or meat	Poultry	20	7	25	19	93	71	14
**C**	**78**	Ab-RW8	Spain	2010	environmental waters	Environmental waters	20	7	20	15	186	8	14
**C**	**78**	LMG14714	Greece	Unk.	Unk.	Unk.	20	7	20	15	186	8	14
**C**	**79**	14181	Thailand	2003	pork offal or meat	Pig	20	7	25	17	41	37	33
**C**	**80**	14251	Thailand	2003	fish	Seafood	20	7	20	23	186	45	37
**C**	**81**	R-8636	Belgium	Unk.	human stool	Human	20	12	11	19	189	127	88
**C**	**82**	14161	Thailand	2003	chicken offal or meat	Poultry	20	18	18	21	28	23	20
**C**	**83**	14195	Vietnam	2002	human stool	Human	20	23	20	23	186	45	37
**C**	**88**	RM4471	USA	Unk.	primate	Human	20	39	4	11	110	63	56
**C**	**90**	RM5226	Turkey	2000	poultry carcass swab	Poultry	20	39	19	15	133	87	58
**C**	**90**	R-14600	Turkey	Unk.	poultry carcass swab	Poultry	20	39	19	15	133	87	58
**C**	**90**	R-14606	Turkey	Unk.	poultry carcass swab	Poultry	20	39	19	15	133	87	58
**C**	**95**	14163	Thailand	2003	chicken offal or meat	Poultry	22	7	1	14	31	25	22
**C**	**99**	14254	Thailand	2003	chicken offal or meat	Poultry	23	7	1	15	89	69	18
**C**	**100**	RM4839	USA	Unk.	poultry carcass swab	Poultry	23	7	11	44	5	90	62
**C**	**101**	RM5225	Turkey	Unk.	poultry carcass swab	Poultry	23	7	41	19	132	101	55
**C**	**102**	RM5563	Unk.	Unk.	pig	Pig	23	7	40	15	175	26	58
**C**	**103**	RM5565	Unk.	Unk.	pig	Pig	23	7	7	15	178	40	58
**C**	**104**	RM5579	Unk.	Unk.	Unk.	Unk.	23	7	40	15	181	102	58
**C**	**105**	RM5218	Nigeria	2000	broiler environment	Poultry	23	41	33	13	129	70	31
**C**	**105**	14255	Thailand	2003	chicken offal or meat	Poultry	23	41	33	13	129	70	31
**C**	**109**	14169	Thailand	2003	chicken offal or meat	Poultry	24	23	22	25	34	28	26
**C**	**113**	14171	Thailand	2003	chicken offal or meat	Poultry	26	24	17	20	67	22	28
**C**	**114**	Ab-FW12	Spain	2010	environmental waters	Environmental waters	27	25	7	2	102	31	29
**C**	**114**	14256	Thailand	2003	chicken offal or meat	Poultry	27	25	7	2	102	31	29
**C**	**115**	14173	Thailand	2003	chicken offal or meat	Poultry	27	25	7	2	15	31	29
**C**	**116**	14178	Thailand	2003	pork offal or meat	Pig	28	5	7	7	167	7	6
**C**	**117**	RM5543	Thailand	Unk.	human stool	Human	28	5	7	7	120	7	6
**C**	**117**	RM5551	Thailand	Unk.	human stool	Human	28	5	7	7	120	7	6
**C**	**117**	14189	Vietnam	2002	human stool	Human	28	5	7	7	120	7	6
**C**	**117**	14207	Thailand	2004	human stool	Human	28	5	7	7	120	7	6
**C**	**117**	14219	Thailand	2003	pork offal or meat	Pig	28	5	7	7	120	7	6
**C**	**119**	Ab-FW11	Spain	2010	environmental waters	Environmental waters	30	5	5	30	120	35	4
**C**	**119**	RM4464	USA	Unk.	human stool	Human	30	5	5	30	120	35	4
**C**	**120**	RM4485	USA	Unk.	turkey	Poultry	30	5	5	30	120	83	58
**C**	**121**	RM5124	USA	2005	chicken offal or meat	Poultry	30	5	5	30	125	35	4

Clade	ST	Strain	Location	Date	Source (PubMLST)	Source (Figure 4)	*aspA*	*atpA*	*glnA*	*gltA*	*glyA*	*pgm*	*tkt*
**C**	**122**	RM5126	USA	2005	chicken offal or meat	Poultry	30	5	5	30	36	35	4
**C**	**122**	RM5133	USA	2005	chicken offal or meat	Poultry	30	5	5	30	36	35	4
**C**	**123**	RM5567	Unk.	Unk.	pig	Pig	30	5	9	19	180	101	55
**C**	**123**	RM5568	Unk.	Unk.	pig	Pig	30	5	9	19	180	101	55
**C**	**123**	RM5591	USA	Unk.	pig	Pig	30	5	9	19	180	101	55
**C**	**123**	RM5596	Unk.	Unk.	pig	Pig	30	5	9	19	180	101	55
**C**	**124**	14184	Vietnam	2001	human stool	Human	30	5	5	30	44	35	4
**C**	**124**	14188	Vietnam	2001	human stool	Human	30	5	5	30	44	35	4
**C**	**138**	RM5523	Unk.	Unk.	human stool	Human	37	5	11	30	144	16	72
**C**	**138**	F72C-1A	USA	2018	environmental waters	Hurricane Florence Water	37	5	11	30	144	16	72
**C**	**138**	34436	UK	2008	cattle	Ruminant	37	5	11	30	144	16	72
**C**	**138**	34437	UK	2008	cattle	Ruminant	37	5	11	30	144	16	72
**C**	**139**	RM4129	South Africa	1997	human stool	Human	37	5	11	17	127	16	40
**C**	**139**	14205	Thailand	2006	human stool	Human	37	5	11	17	127	16	40
**C**	**139**	RM5213	Nigeria	2000	broiler environment	Poultry	37	5	11	17	127	16	40
**C**	**140**	RM3790	South Africa	1997	human stool	Human	37	7	1	23	103	75	33
**C**	**141**	14248	Thailand	2003	squid	Seafood	37	23	26	12	187	65	10
**C**	**156**	14235	Thailand	2003	chicken offal or meat	Poultry	44	38	1	43	144	37	33
**C**	**157**	14250	Thailand	2003	fish	Seafood	44	38	1	12	88	67	33
**C**	**163**	14244	Thailand	2003	pork offal or meat	Pig	47	7	2	44	83	37	47
**C**	**164**	14245	Thailand	2003	pork offal or meat	Pig	48	25	1	19	84	8	6
**C**	**166**	AF-ARCO-70	Thailand	Unk.	human	Human	50	40	19	45	165	68	48
**C**	**166**	14253	Thailand	2003	chicken offal or meat	Poultry	50	40	19	45	165	68	48
**C**	**167**	RM5548	Thailand	Unk.	human stool	Human	50	40	19	12	173	68	48
**C**	**167**	RM5561	Thailand	Unk.	human stool	Human	50	40	19	12	173	68	48
**C**	**169**	14261	Thailand	2003	pork offal or meat	Pig	52	39	11	19	98	58	9
**C**	**175**	RM4594	USA	Unk.	turkey	Poultry	56	2	11	23	114	37	20
**C**	**177**	RM4597	USA	Unk.	turkey	Poultry	58	41	1	15	116	70	6
**C**	**185**	RM5216	Nigeria	2000	broiler environment	Poultry	64	52	30	17	128	20	66
**C**	**190**	RM5229	Turkey	2000	poultry carcass swab	Poultry	69	2	11	23	135	103	70
**C**	**191**	RM5237	UK	1995	poultry carcass swab	Poultry	70	56	29	19	120	105	6
**C**	**195**	Ab-RW10	Spain	2010	environmental waters	Environmental waters	73	2	11	44	146	111	40
**C**	**195**	RM5522	UK	Unk.	pig fetus, aborted	Pig	73	2	11	44	146	111	40
**C**	**195**	RM5524	UK	Unk.	pig fetus, aborted	Pig	73	2	11	44	146	111	40
**C**	**196**	R-700	Germany	Unk.	human stool	Human	73	12	1	65	188	10	9
**C**	**203**	LMG 15577	Netherlands	1994	human stool	Human	80	4	9	12	120	126	4
**C**	**259**	RM5516	Unk.	Unk.	pig	Pig	37	5	11	30	120	16	72

Clade	ST	Strain	Location	Date	Source (PubMLST)	Source (Figure 4)	*aspA*	*atpA*	*glnA*	*gltA*	*glyA*	*pgm*	*tkt*
**C**	**272**	14166	Thailand	Unk.	chicken offal or meat	Poultry	23	21	11	23	32	26	143
**C**	**301**	35160	UK	2008	cattle	Ruminant	37	5	24	17	360	220	40
**C**	**301**	35161	UK	2008	cattle	Ruminant	37	5	24	17	360	220	40
**C**	**301**	35244	UK	2008	cattle	Ruminant	37	5	24	17	360	220	40
**C**	**301**	35245	UK	2008	cattle	Ruminant	37	5	24	17	360	220	40
**C**	**301**	35246	UK	2008	cattle	Ruminant	37	5	24	17	360	220	40
**C**	**301**	35249	UK	2008	cattle	Ruminant	37	5	24	17	360	220	40
**C**	**315**	A61	Belgium	2007	pig	Pig	23	7	40	19	346	40	58
**C**	**315**	B01	Belgium	2007	pig	Pig	23	7	40	19	346	40	58
**C**	**315**	B07	Belgium	2007	pig	Pig	23	7	40	19	346	40	58
**C**	**315**	B10	Belgium	2007	pig	Pig	23	7	40	19	346	40	58
**C**	**315**	B12	Belgium	2007	pig	Pig	23	7	40	19	346	40	58
**C**	**315**	B38	Belgium	2007	pig	Pig	23	7	40	19	346	40	58
**C**	**315**	B39	Belgium	2007	pig	Pig	23	7	40	19	346	40	58
**C**	**318**	B18	Belgium	2007	pig	Pig	5	5	5	5	5	50	4
**C**	**318**	B28	Belgium	2007	pig	Pig	5	5	5	5	5	50	4
**C**	**319**	A64	Belgium	2007	pig	Pig	18	4	5	17	379	58	165
**C**	**319**	B20	Belgium	2007	pig	Pig	18	4	5	17	379	58	165
**C**	**319**	G112	Belgium	2007	pig	Pig	18	4	5	17	379	58	165
**C**	**319**	G126	Belgium	2007	pig	Pig	18	4	5	17	379	58	165
**C**	**319**	G145	Belgium	2007	pig	Pig	18	4	5	17	379	58	165
**C**	**319**	G146	Belgium	2007	pig	Pig	18	4	5	17	379	58	165
**C**	**319**	G149	Belgium	2007	pig	Pig	18	4	5	17	379	58	165
**C**	**319**	G21	Belgium	2007	pig	Pig	18	4	5	17	379	58	165
**C**	**319**	G211	Belgium	2007	pig	Pig	18	4	5	17	379	58	165
**C**	**319**	G221	Belgium	2007	pig	Pig	18	4	5	17	379	58	165
**C**	**319**	G26	Belgium	2007	pig	Pig	18	4	5	17	379	58	165
**C**	**319**	G56	Belgium	2007	pig	Pig	18	4	5	17	379	58	165
**C**	**319**	G71	Belgium	2007	pig	Pig	18	4	5	17	379	58	165
**C**	**319**	G86	Belgium	2007	pig	Pig	18	4	5	17	379	58	165
**C**	**319**	G88	Belgium	2007	pig	Pig	18	4	5	17	379	58	165
**C**	**319**	G95	Belgium	2007	pig	Pig	18	4	5	17	379	58	165
**C**	**320**	B25	Belgium	2007	pig	Pig	20	2	26	12	380	17	2
**C**	**320**	G136	Belgium	2007	pig	Pig	20	2	26	12	380	17	2
**C**	**320**	G144	Belgium	2007	pig	Pig	20	2	26	12	380	17	2
**C**	**320**	G167	Belgium	2007	pig	Pig	20	2	26	12	380	17	2
**C**	**320**	G173	Belgium	2007	pig	Pig	20	2	26	12	380	17	2

Clade	ST	Strain	Location	Date	Source (PubMLST)	Source (Figure 4)	*aspA*	*atpA*	*glnA*	*gltA*	*glyA*	*pgm*	*tkt*
**C**	**320**	G179	Belgium	2007	pig	Pig	20	2	26	12	380	17	2
**C**	**320**	G191	Belgium	2007	pig	Pig	20	2	26	12	380	17	2
**C**	**320**	G62	Belgium	2007	pig	Pig	20	2	26	12	380	17	2
**C**	**336**	35659	UK	2008	cattle	Ruminant	6	7	1	19	365	37	20
**C**	**337**	35678	UK	2008	cattle	Ruminant	15	10	11	19	70	102	20
**C**	**338**	36082	UK	2008	cattle	Ruminant	20	2	1	17	419	20	66
**C**	**339**	36067	UK	2008	cattle	Ruminant	20	2	1	137	416	20	165
**C**	**339**	36068	UK	2008	cattle	Ruminant	20	2	1	137	416	20	165
**C**	**339**	36075	UK	2008	cattle	Ruminant	20	2	1	137	416	20	165
**C**	**340**	35286	UK	2008	cattle	Ruminant	20	2	114	19	359	101	165
**C**	**343**	34724	UK	2008	cattle	Ruminant	20	34	5	19	365	37	20
**C**	**343**	34725	UK	2008	cattle	Ruminant	20	34	5	19	365	37	20
**C**	**343**	34726	UK	2008	cattle	Ruminant	20	34	5	19	365	37	20
**C**	**343**	34727	UK	2008	cattle	Ruminant	20	34	5	19	365	37	20
**C**	**344**	35717	UK	2008	cattle	Ruminant	23	7	114	137	90	20	165
**C**	**353**	34688	UK	2008	cattle	Ruminant	175	2	11	19	176	101	10
**C**	**369**	01.02.19	Denmark	2011	chicken	Poultry	30	34	9	30	144	35	175
**C**	**374**	03.02.01	Denmark	2011	chicken	Poultry	5	5	5	19	120	11	65
**C**	**379**	AB-P6	Spain	2011	chicken offal or meat	Poultry	73	12	1	9	120	10	179
**C**	**381**	AB-P8	Spain	2011	chicken offal or meat	Poultry	20	34	9	5	120	239	2
**C**	**381**	Ab-P9	Spain	2011	chicken offal or meat	Poultry	20	34	9	5	120	239	2
**C**	**384**	AB-P17	Spain	2011	chicken offal or meat	Poultry	30	5	9	30	456	35	4
**C**	**385**	AB-P18	Spain	2011	chicken offal or meat	Poultry	5	5	5	15	461	238	2
**C**	**387**	AB-L2	Spain	2011	other food	Other animal/food	20	12	11	11	458	87	178
**C**	**391**	AB-L11	Spain	2011	other food	Other animal/food	5	5	5	15	451	11	10
**C**	**391**	Ab-L12	Spain	2011	milk	Ruminant	5	5	5	15	451	11	10
**C**	**391**	Ab-L13	Spain	2011	milk	Ruminant	5	5	5	15	451	11	10
**C**	**394**	Ab-C6	Spain	2011	pork offal or meat	Pig	5	5	5	19	472	11	65
**C**	**398**	Ab-M2	Spain	2011	mussel	Seafood	20	12	45	15	5	102	58
**C**	**402**	Ab-M3	Spain	2011	mussel	Seafood	211	147	129	147	34	242	184
**C**	**452**	Ab-PV8	Spain	2015	other food	Other animal/food	58	41	1	15	504	70	6
**C**	**462**	AB-FW3	Spain	2010	environmental waters	Environmental waters	23	7	17	19	496	11	65
**C**	**463**	AB-FW6	Spain	2010	environmental waters	Environmental waters	20	5	1	23	502	111	40
**C**	**467**	AB-FW16	Spain	2010	environmental waters	Environmental waters	31	12	1	44	497	7	33
**C**	**467**	AB-FW24	Spain	2010	environmental waters	Environmental waters	31	12	1	44	497	7	33
**C**	**468**	AB-FW19	Spain	2010	environmental waters	Environmental waters	32	17	17	12	67	22	65
**C**	**470**	4374	Spain	2014	human stool	Human	30	5	7	26	125	102	58

Clade	ST	Strain	Location	Date	Source (PubMLST)	Source (Figure 4)	*aspA*	*atpA*	*glnA*	*gltA*	*glyA*	*pgm*	*tkt*
**C**	**472**	AB-FW26	Spain	2010	environmental waters	Environmental waters	5	5	5	44	8	102	2
**C**	**472**	AB-FW33	Spain	2010	environmental waters	Environmental waters	5	5	5	44	8	102	2
**C**	**473**	AB-FW27	Spain	2010	environmental waters	Environmental waters	47	7	138	165	509	261	31
**C**	**476**	AB-FW30	Spain	2010	environmental waters	Environmental waters	20	12	11	11	511	87	178
**C**	**477**	AB-FW32	Spain	2010	environmental waters	Environmental waters	5	34	9	15	512	102	2
**C**	**478**	425	Spain	2014	human stool	Human	73	12	30	9	220	10	179
**C**	**479**	AB-FW44	Spain	2010	environmental waters	Environmental waters	20	12	11	19	513	127	88
**C**	**482**	19032	Spain	2015	human stool	Human	24	23	22	25	10	86	26
**C**	**483**	F03870	Spain	2014	human stool	Human	30	5	1	65	517	16	9
**C**	**484**	858825	Spain	2014	human stool	Human	73	12	1	9	517	10	9
**C**	**486**	77517	Spain	2015	human stool	Human	73	12	1	65	517	10	9
**C**	**488**	38	Italy	2015	mussel	Seafood	73	12	1	88	517	10	9
**C**	**497**	HJXXIII-8	Spain	2014	human stool	Human	30	5	5	30	525	35	4
**C**	**498**	Ab-RW3	Spain	2010	environmental waters	Environmental waters	245	5	5	23	535	272	212
**C**	**504**	Ab-RW13	Spain	2010	environmental waters	Environmental waters	6	23	4	176	540	111	143
**C**	**507**	Ab-CZ2	Spain	2015	other food	Other animal/food	20	2	11	30	380	123	2
**C**	**508**	Ab-CZ4	Spain	2015	other food	Other animal/food	3	17	17	20	67	267	44
**C**	**509**	Ab-CZ5	Spain	2015	other food	Other animal/food	5	5	5	5	512	102	86
**C**	**526**	Ab-Z4	Spain	2015	other food	Other animal/food	19	17	17	12	551	68	65
**C**	**526**	Ab-Z6	Spain	2015	other food	Other animal/food	19	17	17	12	551	68	65
**C**	**528**	Ab-PV3	Spain	2015	other food	Other animal/food	20	39	17	19	530	26	6
**C**	**529**	Ab-LCH1	Spain	2015	other food	Other animal/food	15	10	11	19	460	35	88
**C**	**540**	11V	Italy	2014	clam	Seafood	8	163	1	72	220	15	59
**C**	**576**	AF-ARCO-71	Thailand	Unk.	human stool	Human	30	5	9	30	120	35	4
**C**	**593**	Ab-E1	Spain	2015	other food	Other animal/food	5	5	9	5	5	50	4
**C**	**594**	Ab-PV13	Spain	2015	other food	Other animal/food	255	5	1	30	603	238	170
**C**	**612**	AF-ARCO-56	Thailand	Unk.	human stool	Human	30	5	5	30	615	50	40
**C**	**612**	AF-ARCO-57	Thailand	Unk.	human stool	Human	30	5	5	30	615	50	40
**C**	**640**	HJXXIII-13	Spain	2016	human stool	Human	30	5	5	65	44	10	9
**C**	**641**	HJXXIII-14	Spain	2016	human stool	Human	30	5	9	30	559	35	4
**C**	**644**	HJXXIII-17	Spain	2016	human stool	Human	24	23	22	25	56	86	26
**C**	**676**	HJXXIII-21	Spain	2017	human stool	Human	73	12	1	9	220	10	179
**C**	**678**	HJXXIII-20	Spain	2017	human stool	Human	50	40	19	12	165	68	48
**C**	**740**	F20C-1A	USA	2018	environmental waters	Hurricane Florence Water	58	41	1	22	129	70	6
**D**	**2**	14126	France	2005	human stool	Human	2	2	10	10	10	11	10
**D**	**2**	R-325	Belgium	Unk.	human stool	Human	2	2	10	10	10	11	10
**D**	**2**	19055	Spain	2014	human stool	Human	2	2	10	10	10	11	10

Clade	ST	Strain	Location	Date	Source (PubMLST)	Source (Figure 4)	*aspA*	*atpA*	*glnA*	*gltA*	*glyA*	*pgm*	*tkt*
**D**	**3**	Ab-CZ9	Spain	2015	other food	Other animal/food	2	2	24	27	112	35	20
**D**	**3**	14179	Thailand	2003	pork offal or meat	Pig	2	2	24	27	112	35	20
**D**	**3**	AF-ARCO-80	Thailand	Unk.	pork offal or meat	Pig	2	2	24	27	112	35	20
**D**	**4**	14185	Vietnam	2001	human stool	Human	2	2	1	15	45	40	10
**D**	**4**	14186	Vietnam	2001	human stool	Human	2	2	1	15	45	40	10
**D**	**4**	14187	Vietnam	2001	human stool	Human	2	2	1	15	45	40	10
**D**	**4**	14193	Vietnam	2001	human stool	Human	2	2	1	15	45	40	10
**D**	**5**	14107	France	2004	human stool	Human	2	2	2	2	11	2	2
**D**	**5**	RM4467	USA	Unk.	primate	Human	2	2	2	2	11	2	2
**D**	**6**	14233	Thailand	2003	chicken offal or meat	Poultry	2	23	24	19	75	60	46
**D**	**17**	14112	France	2004	human stool	Human	4	4	4	4	3	4	89
**D**	**17**	14116	France	2003	human stool	Human	4	4	4	4	3	4	89
**D**	**18**	RM5235	Denmark	2001	turkey	Poultry	4	4	4	4	139	4	89
**D**	**18**	34063	UK	2008	cattle	Ruminant	4	4	4	4	139	4	89
**D**	**18**	34064	UK	2008	cattle	Ruminant	4	4	4	4	139	4	89
**D**	**18**	35166	UK	2008	cattle	Ruminant	4	4	4	4	139	4	89
**D**	**18**	35167	UK	2008	cattle	Ruminant	4	4	4	4	139	4	89
**D**	**18**	35168	UK	2008	cattle	Ruminant	4	4	4	4	139	4	89
**D**	**18**	35180	UK	2008	cattle	Ruminant	4	4	4	4	139	4	89
**D**	**18**	35267	UK	2008	cattle	Ruminant	4	4	4	4	139	4	89
**D**	**18**	35628	UK	2008	cattle	Ruminant	4	4	4	4	139	4	89
**D**	**18**	Ab-CH8	Spain	2015	squid	Seafood	4	4	4	4	139	4	89
**D**	**19**	AB6	Belgium	Unk.	cattle	Ruminant	4	39	51	64	184	125	87
**D**	**19**	AB7	Belgium	Unk.	cattle	Ruminant	4	39	51	64	184	125	87
**D**	**20**	RM5528	USA	Unk.	human stool	Human	4	58	44	13	190	4	89
**D**	**30**	14167	Thailand	2003	chicken offal or meat	Poultry	5	12	7	9	33	7	24
**D**	**30**	AF-ARCO-77	Thailand	Unk.	chicken offal or meat	Poultry	5	12	7	9	33	7	24
**D**	**31**	14172	Thailand	2003	chicken offal or meat	Poultry	5	12	11	26	36	30	24
**D**	**31**	AF-ARCO-78	Thailand	Unk.	chicken offal or meat	Poultry	5	12	11	26	36	30	24
**D**	**32**	14217	Thailand	2003	pork offal or meat	Pig	5	12	31	26	66	7	43
**D**	**33**	14218	Thailand	2003	pork offal or meat	Pig	5	12	11	26	60	30	24
**D**	**34**	14223	Thailand	2003	chicken offal or meat	Poultry	5	12	7	26	66	7	24
**D**	**34**	14225	Thailand	2003	chicken offal or meat	Poultry	5	12	7	26	66	7	24
**D**	**38**	14200	Thailand	2005	human stool	Human	6	12	1	12	49	49	39
**D**	**38**	14203	Thailand	2005	human stool	Human	6	12	1	12	49	49	39
**D**	**39**	RM4840	USA	Unk.	poultry carcass swab	Poultry	6	20	20	17	118	91	23
**D**	**40**	RM5212	Nigeria	2000	broiler environment	Poultry	6	20	20	17	72	26	23

Clade	ST	Strain	Location	Date	Source (PubMLST)	Source (Figure 4)	*aspA*	*atpA*	*glnA*	*gltA*	*glyA*	*pgm*	*tkt*
**D**	**40**	14165	Thailand	2003	chicken offal or meat	Poultry	6	20	20	17	72	26	23
**D**	**41**	14176	Thailand	2003	pork offal or meat	Pig	6	20	20	17	72	26	24
**D**	**42**	14230	Thailand	2003	chicken offal or meat	Poultry	6	20	20	41	72	26	23
**D**	**43**	14177	Thailand	2003	pork offal or meat	Pig	6	26	23	19	39	34	31
**D**	**44**	14236	Thailand	2003	chicken offal or meat	Poultry	6	26	23	19	98	34	31
**D**	**45**	14202	Thailand	2005	human stool	Human	6	34	1	12	120	50	14
**D**	**49**	14128	France	Unk.	human stool	Human	10	9	11	11	12	12	11
**D**	**50**	RM5564	Unk.	Unk.	pig	Pig	10	20	11	19	177	123	11
**D**	**51**	14239	Thailand	2003	chicken offal or meat	Poultry	10	39	1	19	78	61	29
**D**	**53**	RM4463	USA	Unk.	human stool	Human	11	4	1	49	106	78	52
**D**	**55**	14228	Thailand	2003	chicken offal or meat	Poultry	11	12	11	19	70	24	11
**D**	**84**	RM1591	USA	Unk.	turkey	Poultry	20	25	7	2	102	32	2
**D**	**85**	14174	Thailand	2003	chicken offal or meat	Poultry	20	25	7	2	37	32	2
**D**	**86**	RM4470	USA	Unk.	primate	Human	20	27	36	48	109	81	55
**D**	**87**	RM4127	Denmark	Unk.	human stool	Human	20	39	34	19	104	76	51
**D**	**89**	RM4610	USA	Unk.	human stool	Human	20	39	7	11	187	87	55
**D**	**91**	RM5227	Turkey	2000	poultry carcass swab	Poultry	20	39	40	19	134	102	2
**D**	**92**	RM5238	UK	1996	chicken	Poultry	20	57	42	30	140	107	14
**D**	**93**	14162	Thailand	2003	chicken offal or meat	Poultry	21	19	19	22	30	24	21
**D**	**96**	RM4479	USA	Unk.	turkey	Poultry	23	5	24	2	124	35	20
**D**	**96**	RM4482	USA	Unk.	turkey	Poultry	23	5	24	2	124	35	20
**D**	**97**	Ab-FW62	Spain	2011	environmental waters	Environmental waters	23	5	24	44	80	35	55
**D**	**97**	RM5224	Turkey	2000	poultry carcass swab	Poultry	23	5	24	44	80	35	55
**D**	**108**	RM5233	Denmark	2001	chicken offal or meat	Poultry	23	44	24	15	124	55	20
**D**	**108**	RM5236	UK	1995	poultry carcass swab	Poultry	23	44	24	15	124	55	20
**D**	**153**	RM4593	USA	Unk.	turkey	Poultry	42	25	7	52	65	86	14
**D**	**154**	14222	Thailand	2003	chicken offal or meat	Poultry	42	25	7	26	64	26	2
**D**	**158**	14260	Thailand	2003	pork offal or meat	Pig	45	25	7	26	96	26	2
**D**	**159**	14238	Thailand	2003	chicken offal or meat	Poultry	45	25	7	26	98	26	14
**D**	**160**	14252	Thailand	2003	chicken offal or meat	Poultry	45	25	7	26	96	26	14
**D**	**161**	14247	Thailand	2003	pork offal or meat	Pig	46	2	19	6	25	24	11
**D**	**162**	14243	Thailand	2003	chicken offal or meat	Poultry	46	19	19	6	25	24	11
**D**	**187**	RM5219	Nigeria	2000	broiler environment	Poultry	66	12	40	15	130	98	58
**D**	**192**	RM5234	Denmark	2000	duck	Poultry	71	25	7	2	138	106	2
**D**	**193**	Ab-C2	Spain	2011	pork offal or meat	Pig	71	25	7	2	102	106	2
**D**	**193**	RM5239	Sweden	1999	sheep	Ruminant	71	25	7	2	102	106	2
**D**	**193**	RM5240	Sweden	1999	sheep	Ruminant	71	25	7	2	102	106	2

Clade	ST	Strain	Location	Date	Source (PubMLST)	Source (Figure 4)	*aspA*	*atpA*	*glnA*	*gltA*	*glyA*	*pgm*	*tkt*
**D**	**193**	RM5241	Sweden	1999	sheep	Ruminant	71	25	7	2	102	106	2
**D**	**198**	RM5527	USA	Unk.	human unspecified	Human	75	25	30	26	65	26	74
**D**	**258**	G168	Belgium	2007	pig	Pig	10	20	11	19	378	123	11
**D**	**258**	G194	Belgium	2007	pig	Pig	10	20	11	19	378	123	11
**D**	**258**	G195	Belgium	2007	pig	Pig	10	20	11	19	378	123	11
**D**	**258**	G30	Belgium	2007	pig	Pig	10	20	11	19	378	123	11
**D**	**293**	34439	UK	2008	cattle	Ruminant	4	4	4	4	148	214	89
**D**	**293**	34440	UK	2008	cattle	Ruminant	4	4	4	4	148	214	89
**D**	**293**	34441	UK	2008	cattle	Ruminant	4	4	4	4	148	214	89
**D**	**293**	34442	UK	2008	cattle	Ruminant	4	4	4	4	148	214	89
**D**	**294**	G05	Belgium	2007	pig	Pig	4	4	4	4	216	4	89
**D**	**294**	G193	Belgium	2007	pig	Pig	4	4	4	4	216	4	89
**D**	**294**	G38	Belgium	2007	pig	Pig	4	4	4	4	216	4	89
**D**	**294**	G47	Belgium	2007	pig	Pig	4	4	4	4	216	4	89
**D**	**294**	G63	Belgium	2007	pig	Pig	4	4	4	4	216	4	89
**D**	**294**	G79	Belgium	2007	pig	Pig	4	4	4	4	216	4	89
**D**	**294**	G80	Belgium	2007	pig	Pig	4	4	4	4	216	4	89
**D**	**294**	34709	UK	2008	cattle	Ruminant	4	4	4	4	216	4	89
**D**	**294**	34716	UK	2008	cattle	Ruminant	4	4	4	4	216	4	89
**D**	**294**	35158	UK	2008	cattle	Ruminant	4	4	4	4	216	4	89
**D**	**295**	31153	UK	2007	cattle	Ruminant	4	4	4	4	346	4	89
**D**	**295**	31162	UK	2007	cattle	Ruminant	4	4	4	4	346	4	89
**D**	**296**	34427	UK	2008	cattle	Ruminant	4	4	4	4	347	214	89
**D**	**296**	34430	UK	2008	cattle	Ruminant	4	4	4	4	347	214	89
**D**	**296**	34431	UK	2008	cattle	Ruminant	4	4	4	4	347	214	89
**D**	**296**	34432	UK	2008	cattle	Ruminant	4	4	4	4	347	214	89
**D**	**296**	34433	UK	2008	cattle	Ruminant	4	4	4	4	347	214	89
**D**	**297**	30878	UK	2007	cattle	Ruminant	4	4	51	126	341	211	89
**D**	**298**	34304	UK	2008	cattle	Ruminant	4	12	1	19	354	213	160
**D**	**298**	34305	UK	2008	cattle	Ruminant	4	12	1	19	354	213	160
**D**	**298**	34306	UK	2008	cattle	Ruminant	4	12	1	19	354	213	160
**D**	**299**	30852	UK	2007	cattle	Ruminant	4	39	51	125	340	125	52
**D**	**299**	30872	UK	2007	cattle	Ruminant	4	39	51	125	340	125	52
**D**	**303**	35222	UK	2008	cattle	Ruminant	150	4	1	122	370	194	52
**D**	**304**	34031	UK	2008	cattle	Ruminant	150	39	40	19	355	214	52
**D**	**305**	31141	UK	2007	cattle	Ruminant	153	4	40	125	345	211	140
**D**	**306**	34423	UK	2008	cattle	Ruminant	153	4	40	125	350	102	9

Clade	ST	Strain	Location	Date	Source (PubMLST)	Source (Figure 4)	*aspA*	*atpA*	*glnA*	*gltA*	*glyA*	*pgm*	*tkt*
**D**	**306**	34424	UK	2008	cattle	Ruminant	153	4	40	125	350	102	9
**D**	**306**	34425	UK	2008	cattle	Ruminant	153	4	40	125	350	102	9
**D**	**306**	34426	UK	2008	cattle	Ruminant	153	4	40	125	350	102	9
**D**	**308**	31387	UK	2007	cattle	Ruminant	169	4	4	4	350	209	89
**D**	**308**	31402	UK	2007	cattle	Ruminant	169	4	4	4	350	209	89
**D**	**308**	31790	UK	2007	cattle	Ruminant	169	4	4	4	350	209	89
**D**	**308**	31794	UK	2007	cattle	Ruminant	169	4	4	4	350	209	89
**D**	**308**	31791	UK	2007	cattle	Ruminant	169	4	4	4	350	209	89
**D**	**308**	31795	UK	2007	cattle	Ruminant	169	4	4	4	350	209	89
**D**	**308**	31792	UK	2007	cattle	Ruminant	169	4	4	4	350	209	89
**D**	**308**	31796	UK	2007	cattle	Ruminant	169	4	4	4	350	209	89
**D**	**308**	31793	UK	2007	cattle	Ruminant	169	4	4	4	350	209	89
**D**	**308**	31797	UK	2007	cattle	Ruminant	169	4	4	4	350	209	89
**D**	**308**	31798	UK	2007	cattle	Ruminant	169	4	4	4	350	209	89
**D**	**308**	34446	UK	2008	cattle	Ruminant	169	4	4	4	350	209	89
**D**	**308**	36099	UK	2008	cattle	Ruminant	169	4	4	4	350	209	89
**D**	**308**	36100	UK	2008	cattle	Ruminant	169	4	4	4	350	209	89
**D**	**311**	35184	UK	2008	cattle	Ruminant	177	12	1	23	368	214	159
**D**	**316**	B04	Belgium	2007	pig	Pig	4	12	51	125	376	11	87
**D**	**317**	B14	Belgium	2007	pig	Pig	37	23	1	30	377	55	55
**D**	**317**	B15	Belgium	2007	pig	Pig	37	23	1	30	377	55	55
**D**	**317**	B19	Belgium	2007	pig	Pig	37	23	1	30	377	55	55
**D**	**317**	B48	Belgium	2007	pig	Pig	37	23	1	30	377	55	55
**D**	**321**	B27	Belgium	2007	pig	Pig	20	39	11	19	134	102	2
**D**	**323**	G132	Belgium	2007	pig	Pig	10	20	20	19	382	123	11
**D**	**323**	G41	Belgium	2007	pig	Pig	10	20	20	19	382	123	11
**D**	**323**	G51	Belgium	2007	pig	Pig	10	20	20	19	382	123	11
**D**	**323**	G77	Belgium	2007	pig	Pig	10	20	20	19	382	123	11
**D**	**323**	G83	Belgium	2007	pig	Pig	10	20	20	19	382	123	11
**D**	**323**	G90	Belgium	2007	pig	Pig	10	20	20	19	382	123	11
**D**	**324**	B16	Belgium	2007	pig	Pig	10	20	20	19	378	123	11
**D**	**326**	103	Belgium	2007	pig	Pig	4	12	1	19	387	213	159
**D**	**326**	135	Belgium	2007	pig	Pig	4	12	1	19	387	213	159
**D**	**328**	36080	UK	2008	cattle	Ruminant	4	4	1	19	418	11	169
**D**	**329**	35716	UK	2008	cattle	Ruminant	4	4	40	19	414	11	87
**D**	**330**	36078	UK	2008	cattle	Ruminant	4	4	40	19	418	11	169
**D**	**330**	36079	UK	2008	cattle	Ruminant	4	4	40	19	418	11	169

Clade	ST	Strain	Location	Date	Source (PubMLST)	Source (Figure 4)	*aspA*	*atpA*	*glnA*	*gltA*	*glyA*	*pgm*	*tkt*
**D**	**331**	36110	UK	2008	cattle	Ruminant	4	4	44	128	408	211	163
**D**	**331**	36111	UK	2008	cattle	Ruminant	4	4	44	128	408	211	163
**D**	**331**	36112	UK	2008	cattle	Ruminant	4	4	44	128	408	211	163
**D**	**331**	36113	UK	2008	cattle	Ruminant	4	4	44	128	408	211	163
**D**	**332**	36069	UK	2008	cattle	Ruminant	4	4	44	138	415	11	158
**D**	**332**	36095	UK	2008	cattle	Ruminant	4	4	44	138	415	11	158
**D**	**333**	35758	UK	2008	cattle	Ruminant	4	4	51	126	341	221	89
**D**	**334**	31894	UK	2007	cattle	Ruminant	4	12	1	19	343	213	159
**D**	**335**	36106	UK	2008	cattle	Ruminant	4	39	51	125	65	126	87
**D**	**335**	36108	UK	2008	cattle	Ruminant	4	39	51	125	65	126	87
**D**	**335**	36109	UK	2008	cattle	Ruminant	4	39	51	125	65	126	87
**D**	**341**	35982	UK	2008	cattle	Ruminant	20	4	40	19	214	228	169
**D**	**341**	35983	UK	2008	cattle	Ruminant	20	4	40	19	214	228	169
**D**	**342**	35601	UK	2008	cattle	Ruminant	20	12	44	23	366	219	168
**D**	**347**	30567	UK	2007	cattle	Ruminant	150	4	1	122	353	194	52
**D**	**347**	30618	UK	2007	cattle	Ruminant	150	4	1	122	353	194	52
**D**	**348**	35660	UK	2008	cattle	Ruminant	153	4	40	19	411	211	9
**D**	**349**	36091	UK	2008	cattle	Ruminant	153	4	40	138	410	11	158
**D**	**350**	36107	UK	2008	cattle	Ruminant	153	4	44	123	345	211	158
**D**	**351**	36104	UK	2008	cattle	Ruminant	153	136	51	125	413	125	52
**D**	**351**	36105	UK	2008	cattle	Ruminant	153	136	51	125	413	125	52
**D**	**352**	35265	UK	2008	cattle	Ruminant	175	2	11	17	389	211	158
**D**	**352**	35266	UK	2008	cattle	Ruminant	175	2	11	17	389	211	158
**D**	**352**	35268	UK	2008	cattle	Ruminant	175	2	11	17	389	211	158
**D**	**354**	35898	UK	2008	cattle	Ruminant	177	4	40	19	340	211	14
**D**	**354**	35899	UK	2008	cattle	Ruminant	177	4	40	19	340	211	14
**D**	**354**	35900	UK	2008	cattle	Ruminant	177	4	40	19	340	211	14
**D**	**354**	35901	UK	2008	cattle	Ruminant	177	4	40	19	340	211	14
**D**	**355**	31711	UK	2007	cattle	Ruminant	177	39	40	127	351	63	158
**D**	**355**	31712	UK	2007	cattle	Ruminant	177	39	40	127	351	63	158
**D**	**355**	31713	UK	2007	cattle	Ruminant	177	39	40	127	351	63	158
**D**	**356**	35687	UK	2008	cattle	Ruminant	182	4	40	125	413	125	52
**D**	**359**	Ab-P4	Spain	2011	chicken offal or meat	Poultry	48	25	41	19	440	102	2
**D**	**360**	Ab-L7	Spain	2011	milk	Ruminant	20	39	34	19	441	26	51
**D**	**367**	01.02.02	Denmark	2011	chicken	Poultry	153	4	11	11	177	102	11
**D**	**367**	01.02.03	Denmark	2011	chicken	Poultry	153	4	11	11	177	102	11
**D**	**367**	01.02.13	Denmark	2011	chicken	Poultry	153	4	11	11	177	102	11

Clade	ST	Strain	Location	Date	Source (PubMLST)	Source (Figure 4)	*aspA*	*atpA*	*glnA*	*gltA*	*glyA*	*pgm*	*tkt*
**D**	**367**	03.02.01.01	Denmark	2011	chicken	Poultry	153	4	11	11	177	102	11
**D**	**367**	03.02.07	Denmark	2011	chicken	Poultry	153	4	11	11	177	102	11
**D**	**367**	03.02.09	Denmark	2011	chicken	Poultry	153	4	11	11	177	102	11
**D**	**367**	03.02.10	Denmark	2011	chicken	Poultry	153	4	11	11	177	102	11
**D**	**367**	03.02.11	Denmark	2011	chicken	Poultry	153	4	11	11	177	102	11
**D**	**367**	03.02.12	Denmark	2011	chicken	Poultry	153	4	11	11	177	102	11
**D**	**367**	03.02.13	Denmark	2011	chicken	Poultry	153	4	11	11	177	102	11
**D**	**367**	03.02.16	Denmark	2011	chicken	Poultry	153	4	11	11	177	102	11
**D**	**367**	03.02.17	Denmark	2011	chicken	Poultry	153	4	11	11	177	102	11
**D**	**367**	03.02.19	Denmark	2011	chicken	Poultry	153	4	11	11	177	102	11
**D**	**367**	03.02.20	Denmark	2011	chicken	Poultry	153	4	11	11	177	102	11
**D**	**367**	03.03.01	Denmark	2011	chicken	Poultry	153	4	11	11	177	102	11
**D**	**368**	01.02.14	Denmark	2011	chicken	Poultry	5	12	122	15	36	86	2
**D**	**372**	03.02.03	Denmark	2011	chicken	Poultry	23	44	24	14	450	101	20
**D**	**375**	01.02.08.02	Denmark	2011	chicken	Poultry	20	25	7	2	354	32	2
**D**	**376**	03.02.18.01	Denmark	2011	chicken	Poultry	175	25	41	19	88	101	14
**D**	**376**	03.02.18.02	Denmark	2011	chicken	Poultry	175	25	41	19	88	101	14
**D**	**386**	AB-L1	Spain	2011	other food	Other animal/food	20	39	34	19	460	87	51
**D**	**388**	AB-L3	Spain	2011	other food	Other animal/food	20	39	34	19	460	234	51
**D**	**388**	Ab-L4	Spain	2011	milk	Ruminant	20	39	34	19	460	234	51
**D**	**388**	Ab-L5	Spain	2011	milk	Ruminant	20	39	34	19	460	234	51
**D**	**388**	Ab-L6	Spain	2011	milk	Ruminant	20	39	34	19	460	234	51
**D**	**389**	AB-L8	Spain	2011	other food	Other animal/food	182	58	4	146	185	236	160
**D**	**390**	AB-L10	Spain	2011	other food	Other animal/food	10	145	124	19	454	102	11
**D**	**390**	Ab-L14	Spain	2011	milk	Ruminant	10	145	124	19	454	102	11
**D**	**393**	Ab-A7	Spain	2011	clam	Seafood	4	4	4	4	468	4	185
**D**	**397**	Ab-A4	Spain	2011	clam	Seafood	15	10	5	15	36	243	2
**D**	**397**	Ab-A5	Spain	2011	clam	Seafood	15	10	5	15	36	243	2
**D**	**399**	Ab-C4	Spain	2011	pork offal or meat	Pig	20	39	34	19	470	102	51
**D**	**400**	Ab-C5	Spain	2011	pork offal or meat	Pig	44	150	11	23	471	245	187
**D**	**401**	Ab-C3	Spain	2011	pork offal or meat	Pig	153	4	7	19	467	102	158
**D**	**466**	AB-FW15	Spain	2010	environmental waters	Environmental waters	213	44	24	15	124	55	20
**D**	**475**	AB-FW29	Spain	2010	environmental waters	Environmental waters	231	133	51	23	510	4	160
**D**	**475**	Ab-BER4	Spain	2015	other food	Other animal/food	231	133	51	23	510	4	160
**D**	**489**	39	Italy	2015	mussel	Seafood	73	12	1	9	32	263	14
**D**	**493**	HSJR-2	Spain	2013	human stool	Human	6	34	1	12	522	50	55
**D**	**506**	Ab-CZ1	Spain	2015	other food	Other animal/food	4	12	1	19	418	213	159

Clade	ST	Strain	Location	Date	Source (PubMLST)	Source (Figure 4)	*aspA*	*atpA*	*glnA*	*gltA*	*glyA*	*pgm*	*tkt*
**D**	**510**	Ab-CZ6	Spain	2015	other food	Other animal/food	23	44	24	44	112	35	20
**D**	**510**	Ab-CZ10	Spain	2015	other food	Other animal/food	23	44	24	44	112	35	20
**D**	**512**	Ab-G2	Spain	2015	shrimp	Seafood	4	164	40	23	528	268	4
**D**	**512**	Ab-CH1	Spain	2015	squid	Seafood	4	164	40	23	528	268	4
**D**	**512**	Ab-CH11	Spain	2015	squid	Seafood	4	164	40	23	528	268	4
**D**	**513**	Ab-BER7	Spain	2015	other food	Other animal/food	4	165	40	19	351	211	4
**D**	**513**	Ab-G3	Spain	2015	shrimp	Seafood	4	165	40	19	351	211	4
**D**	**513**	Ab-CH6	Spain	2015	squid	Seafood	4	165	40	19	351	211	4
**D**	**513**	Ab-CH12	Spain	2015	squid	Seafood	4	165	40	19	351	211	4
**D**	**514**	Ab-PV4	Spain	2015	other food	Other animal/food	45	25	20	15	531	102	2
**D**	**518**	Ab-CH10	Spain	2015	squid	Seafood	231	133	51	23	510	211	160
**D**	**519**	Ab-BER6	Spain	2015	other food	Other animal/food	240	133	40	12	541	278	160
**D**	**520**	Ab-PV6	Spain	2015	other food	Other animal/food	242	20	20	31	532	102	209
**D**	**520**	Ab-PV7	Spain	2015	Unk.	Unk.	242	20	20	31	532	102	209
**D**	**525**	Ab-PV14	Spain	2015	other food	Other animal/food	153	4	11	15	545	102	2
**D**	**533**	Ab-QS1	Spain	2015	other food	Other animal/food	10	145	124	19	549	102	11
**D**	**536**	Ab-PV15	Spain	2015	other food	Other animal/food	23	5	24	27	80	295	20
**D**	**538**	20/O	Italy	2015	other food	Other animal/food	13	4	40	15	521	244	58
**D**	**587**	Ab-CZ3	Spain	2015	other food	Other animal/food	5	12	122	15	67	26	2
**D**	**590**	Ab-Z7	Spain	2015	other food	Other animal/food	231	133	51	19	510	213	160
**D**	**620**	AF-ARCO-69	Thailand	Unk.	human stool	Human	42	25	7	44	616	332	2
**D**	**645**	HJXXIII-18	Spain	2016	human stool	Human	73	12	1	9	385	263	14
**D**	**677**	HJXXIII-19	Spain	2016	human stool	Human	15	10	34	23	441	368	51
*******	**1**	RM4018^T	USA	1990	human stool	Human	1	1	1	1	1	1	1
*******	**22**	14144	Thailand	2003	beef offal or meat	Ruminant	5	5	12	12	83	13	12
*******	**25**	14175	Thailand	2003	pork offal or meat	Pig	5	5	1	12	83	33	30
*******	**27**	14227	Thailand	2003	chicken offal or meat	Poultry	5	5	24	37	68	2	20
*******	**28**	14240	Thailand	2003	chicken offal or meat	Poultry	5	5	24	27	79	62	20
*******	**28**	14241	Thailand	2003	chicken offal or meat	Poultry	5	5	24	27	79	62	20
*******	**29**	14246	Thailand	2003	pork offal or meat	Pig	5	5	1	12	83	64	12
*******	**47**	14124	France	2003	human stool	Human	8	8	8	8	9	9	8
*******	**62**	RM4848	USA	Unk.	pork offal or meat	Pig	14	49	26	55	123	92	63
*******	**98**	14242	Thailand	2003	chicken offal or meat	Poultry	23	5	24	2	81	63	20
*******	**106**	RM4480	USA	Unk.	turkey	Poultry	23	44	24	2	170	62	20
*******	**106**	RM4481	USA	Unk.	turkey	Poultry	23	44	24	2	170	62	20
*******	**106**	RM4484	USA	Unk.	turkey	Poultry	23	44	24	2	170	62	20
*******	**107**	RM5231	Denmark	2001	duck	Poultry	23	44	24	2	137	62	20

Clade	ST	Strain	Location	Date	Source (PubMLST)	Source (Figure 4)	*aspA*	*atpA*	*glnA*	*gltA*	*glyA*	*pgm*	*tkt*
***	**168**	14258	Thailand	2003	pork offal or meat	Pig	51	16	1	15	94	26	49
***	**172**	RM4592	USA	Unk.	turkey	Poultry	55	45	26	48	113	85	59
***	**174**	RM5537	USA	Unk.	human stool	Human	55	65	48	55	158	38	81
***	**182**	RM4851	USA	Unk.	turkey	Poultry	62	12	39	56	124	94	23
***	**182**	RM4852	USA	Unk.	turkey	Poultry	62	12	39	56	124	94	23
***	**183**	RM5209	Nigeria	2000	broiler environment	Poultry	62	12	7	56	124	96	23
***	**200**	RM5534	USA	Unk.	human blood culture	Human	77	10	24	17	19	117	20
***	**267**	RM4850	USA	Unk.	horse	Other animal/food	14	49	26	55	144	92	13
***	**310**	34042	UK	2008	cattle	Ruminant	171	8	8	8	356	216	164
***	**310**	34044	UK	2008	cattle	Ruminant	171	8	8	8	356	216	164
***	**380**	AB-P7	Spain	2011	chicken offal or meat	Poultry	210	65	125	144	457	240	180
***	**382**	AB-P13	Spain	2011	chicken offal or meat	Poultry	23	7	26	15	459	102	158
***	**395**	Ab-V1	Spain	2011	beef offal or meat	Ruminant	5	5	127	24	463	107	63
***	**465**	AB-FW14	Spain	2010	environmental waters	Environmental waters	60	15	24	27	102	62	20
***	**469**	AB-FW23	Spain	2010	environmental waters	Environmental waters	23	5	45	37	498	56	20
***	**480**	AB-FW57	Spain	2010	environmental waters	Environmental waters	232	8	137	166	514	262	204
***	**487**	34	Italy	2015	mussel	Seafood	65	65	125	144	120	240	180
***	**490**	HJXXIII-2	Spain	2013	human stool	Human	237	45	26	48	113	85	205
***	**491**	HJXXIII-3	Spain	2013	human stool	Human	8	8	137	166	519	19	204
***	**503**	Ab-RW12	Spain	2010	environmental waters	Environmental waters	243	166	137	171	539	270	210
***	**516**	Ab-PV11	Spain	2015	other food	Other animal/food	254	171	1	12	544	275	223
***	**516**	Ab-PV12	Spain	2015	other food	Other animal/food	254	171	1	12	544	275	223
***	**517**	Ab-BER1	Spain	2015	other food	Other animal/food	65	65	125	144	527	240	180
***	**517**	Ab-BER2	Spain	2015	other food	Other animal/food	65	65	125	144	527	240	180
***	**517**	Ab-BER3	Spain	2015	other food	Other animal/food	65	65	125	144	527	240	180
***	**517**	Ab-G1	Spain	2015	shrimp	Seafood	65	65	125	144	527	240	180
***	**517**	Ab-CH7	Spain	2015	squid	Seafood	65	65	125	144	527	240	180
***	**517**	Ab-CH9	Spain	2015	squid	Seafood	65	65	125	144	527	240	180
***	**517**	Ab-CH5	Spain	2015	squid	Seafood	65	65	125	144	527	240	180
***	**524**	Ab-PV10	Spain	2015	other food	Other animal/food	81	15	154	179	543	274	222
***	**588**	Ab-Z1	Spain	2015	other food	Other animal/food	74	8	24	60	191	112	73
***	**595**	Ab-PV16	Spain	2015	Unk.	Unk.	60	15	15	140	178	238	2
***	**675**	55	Italy	2016	mussel	Seafood	332	8	1	166	685	367	292
